# The increase in cardiac output induced by a decrease in positive end-expiratory pressure reliably detects volume responsiveness: the PEEP-test study

**DOI:** 10.1186/s13054-023-04424-7

**Published:** 2023-04-09

**Authors:** Christopher Lai, Rui Shi, Alexandra Beurton, Francesca Moretto, Soufia Ayed, Nicolas Fage, Francesco Gavelli, Arthur Pavot, Martin Dres, Jean-Louis Teboul, Xavier Monnet

**Affiliations:** 1 AP-HP, Service de médecine intensive-réanimation, Hôpitaux Universitaires Paris-Saclay, Hôpital de Bicêtre, DMU CORREVE, Inserm UMR S_999, FHU SEPSIS, Groupe de Recherche Clinique CARMAS, Université Paris-Saclay, 78 Rue du Général Leclerc, 94270 Le Kremlin-Bicêtre, France; 2 Service de Médecine intensive - Réanimation, AP-HP, Groupe Hospitalier Universitaire APHP-Sorbonne Université, Site Pitié-Salpêtrière, Paris, France; 3 INSERM, UMRS_1158 Neurophysiologie Respiratoire Expérimentale et Clinique, Sorbonne Université, Paris, France

**Keywords:** ARDS, Fluid responsiveness, Hemodynamic monitoring, Passive leg raising, Shock

## Abstract

**Background:**

In patients on mechanical ventilation, positive end-expiratory pressure (PEEP) can decrease cardiac output through a decrease in cardiac preload and/or an increase in right ventricular afterload. Increase in central blood volume by fluid administration or passive leg raising (PLR) may reverse these phenomena through an increase in cardiac preload and/or a reopening of closed lung microvessels. We hypothesized that a transient decrease in PEEP (PEEP-test) may be used as a test to detect volume responsiveness.

**Methods:**

Mechanically ventilated patients with PEEP ≥ 10 cmH_2_O (“high level”) and without spontaneous breathing were prospectively included. Volume responsiveness was assessed by a positive PLR-test, defined as an increase in pulse-contour-derived cardiac index (CI) during PLR ≥ 10%. The PEEP-test consisted in reducing PEEP from the high level to 5 cmH_2_O for one minute. Pulse-contour-derived CI (PiCCO2) was monitored during PLR and the PEEP-test.

**Results:**

We enrolled 64 patients among whom 31 were volume responsive. The median increase in CI during PLR was 14% (11–16%). The median PEEP at baseline was 12 (10–15) cmH_2_O and the PEEP-test resulted in a median decrease in PEEP of 7 (5–10) cmH_2_O, without difference between volume responsive and unresponsive patients. Among volume responsive patients, the PEEP-test induced a significant increase in CI of 16% (12–20%) (from 2.4 ± 0.7 to 2.9 ± 0.9 L/min/m^2^, *p* < 0.0001) in comparison with volume unresponsive patients. In volume unresponsive patients, PLR and the PEEP-test increased CI by 2% (1–5%) and 6% (3–8%), respectively. Volume responsiveness was predicted by an increase in CI > 8.6% during the PEEP-test with a sensitivity of 96.8% (95% confidence interval (95%CI): 83.3–99.9%) and a specificity of 84.9% (95%CI 68.1–94.9%). The area under the receiver operating characteristic curve of the PEEP-test for detecting volume responsiveness was 0.94 (95%CI 0.85–0.98) (*p* < 0.0001 *vs.* 0.5). Spearman’s correlation coefficient between the changes in CI induced by PLR and the PEEP-test was 0.76 (95%CI 0.63–0.85, *p* < 0.0001).

**Conclusions:**

A CI increase > 8.6% during a PEEP-test, which consists in reducing PEEP to 5 cmH_2_O, reliably detects volume responsiveness in mechanically ventilated patients with a PEEP ≥ 10 cmH_2_O.

*Trial registration* ClinicalTrial.gov (NCT 04,023,786). Registered July 18, 2019. Ethics Committee approval CPP Est III (N° 2018-A01599-46).

**Supplementary Information:**

The online version contains supplementary material available at 10.1186/s13054-023-04424-7.

## Introduction

In patients with acute circulatory failure, predicting volume responsiveness before deciding to infuse or not a fluid bolus, as recommended by international guidelines [1, 2], should avoid unnecessary fluid administration. For this purpose, several dynamic tests and indices have been developed [3].

However, pulse pressure variation (PPV) and stroke volume variation (SVV) have strict conditions of validity that limit their generalizability [4, 5]. The reliability of the distensibility of the inferior and superior vena cava has been questioned [6–8]. Passive leg raising (PLR), which acts as a reversible volume challenge [9–11], might sometimes be cumbersome. In patients under mechanical ventilation, the end-expiratory occlusion test requires a rather long respiratory hold, which may be interrupted by some patients with strong breathing activity [12]. Thus, there may be a place for other easy-to-perform and widely applicable tests of volume responsiveness.

In mechanically ventilated patients, positive end-expiratory pressure (PEEP) has a two-fold effect [13]: on the one hand it may increase the pulmonary vascular resistance and the right ventricular afterload by increasing the transpulmonary pressure, and on the other hand by increasing the intrathoracic pressure, it may decrease cardiac preload [14, 15]. Considering the latter effect, a transient increase in PEEP was proposed as a volume responsiveness test in ventilated patients without low lung compliance and a low PEEP level [16, 17]. However, in patients ventilated with high levels of PEEP and low lung compliance, the test reliability may be impaired, because increasing PEEP further may significantly increase the right ventricular afterload [14], altering the effects of PEEP changes on cardiac output. For testing volume responsiveness, decreasing PEEP may better mimic a preload challenge.

The goal of this study, conducted in critically ill patients receiving low tidal volume ventilation with a PEEP level ≥ 10 cmH_2_O, was to evaluate if a PEEP-test, consisting of a transient decrease in PEEP from a high to a low level (5 cmH_2_O), might accurately detect volume responsiveness, defined as a positive PLR-test.

## Patients and methods

### Patients

This prospective interventional study was conducted between January 3, 2020, and September 30, 2021, in the intensive care unit of two tertiary hospitals (Bicêtre and Pitié-Salpêtrière hospitals, Paris, France). Our study was approved by the Comité de Protection des Personnes Est-III (2018-A01599-46) and registered on ClinicalTrials.gov (NCT 04,023,786). It was conducted according to the STARD guidelines [18] (Additional file 1: Appendix 1).

Written informed consent was obtained from all patients, their next of kin or another surrogate decision maker, as appropriate. If patients or surrogate decision maker could not provide informed consent, post-hoc consent was obtained from patients who survived.

We included patients ≥ 18 years old, on invasive mechanical ventilation without spontaneous breathing, with PEEP ≥ 10 cmH_2_O, with pulse oxygen saturation (SpO_2_) ≥ 90%, monitored by a transpulmonary thermodilution device (PiCCO2, Pulsion Medical Systems, Getinge, Feldkirchen, Germany) and in whom attending physicians decided to assess volume responsiveness. Exclusion criteria were extracorporeal membrane oxygenation, venous compression stockings, contraindication to performing PLR, and a decrease in SpO_2_ under 80% during the PEEP-test. Non-inclusion criteria were refusal to participate in the study and unavailability of the investigators.

### Mechanical ventilation

Patients were ventilated in the volume assist-control mode (Evita 4 or V500, Dräger, Lübeck, Germany). By default, the tidal volume was set at 6 mL/kg of predicted body weight. PEEP was set by the clinicians in charge, with the goal of keeping the plateau pressure ≤ 30 cmH_2_O. Respiratory mechanics were assessed while the patient was passively ventilated (no triggering observed on the airway pressure curve). Plateau pressure was measured during a 0.2 s end-inspiratory occlusion. Intrinsic PEEP was measured during an end-expiratory occlusion. Compliance of the respiratory system (Crs) was calculated as driving pressure divided by tidal volume.

In patients with ARDS, at the same day as the PEEP-test, we collected the recruitment-to-inflation (R/I) ratio, an estimate of lung recruitability [19]. To calculate it, we measured the volume exhaled during one drop of PEEP of 10 cmH_2_O starting from the set PEEP level. Compliance of the recruited lung between the higher and lower levels or the airway opening pressure was calculated [19]. Airway opening pressure was defined as the elastic airway pressure at which gas volume delivered to a patient became 4 mL greater than the volume compressed in an occluded circuit [19]. Calculation of the R/I ratio was done with the calculator available at www.rtmaven.com. An R/I ratio of 1.0 or more indicates that the likelihood of recruitment is similar or higher, respectively, compared to inflation/hyperinflation [19].

### Hemodynamic measurements

Patients were equipped with a thermistor-tipped arterial femoral catheter and an internal jugular vein catheter, as required by the PiCCO2 device [20]. Pressure sensors were fixed on the upper arm and referenced to the right atrium [21]. The beat-per-beat estimation of stroke volume was performed by pulse contour analysis with the PiCCO2 device [22]. For transpulmonary thermodilution, the result from three consecutive injections of normal saline was averaged [23].

### Study design

After inclusion (Baseline 1), we collected the hemodynamic variables, including heart rate, arterial pressure including pulse pressure (PP), PPV, SVV, central venous pressure, intra-abdominal pressure (IAP), ventilatory parameters, and cardiac index (CI) measured by transpulmonary thermodilution.

Volume responsiveness was assessed by a 1-min PLR-test [10], which was deemed as positive if the pulse contour analysis-derived CI increased by ≥ 10% [9]. After returning to the semi-recumbent position and once CI was stabilized (Baseline 2), the hemodynamic variables were collected again as at Baseline 1, except that CI was measured by pulse contour analysis.

Then, we performed the PEEP-test by reducing PEEP from its baseline level (≥ 10 cmH_2_O) to 5 cmH_2_O for one minute. All hemodynamic variables were collected again, including CI measured from pulse contour analysis. The maximal value of CI during the PEEP-test was collected. PEEP was then increased back to its baseline level. All hemodynamic variables were once again collected after their stabilization (Baseline 3).

In volume responsive patients, and when deemed necessary by the clinicians in charge, fluid bolus (500 mL of normal saline) was administered. In such cases, transpulmonary thermodilution measurements were performed immediately after fluid administration.

Continuous hemodynamic variables, including pulse contour analysis-derived CI, were recorded using PiCCOWin 4.0 software (Pulsion Medical Systems, Feldkirchen, Germany).

### Data analysis

The distribution of continuous variables was tested by the Shapiro–Wilk test. The variables were expressed as mean ± standard deviation, median (interquartile range) or number (percentage). The comparison of variables between different study times was performed with the paired Student's t-test or the Mann–Whitney test, depending on data distribution. Variables in volume responders and non-responders were compared using the Fisher’s exact test or the Wilcoxon test, depending on data distribution.

Receiver operating characteristic (ROC) curves (with 95% confidence interval) were generated to describe the ability to detect volume responsiveness of the following variables: the PEEP-test-induced percent changes of CI (∆CI), PPV (∆PPV), arterial pulse pressure (∆PP) and stroke volume. For each variable, an optimal threshold value was determined to maximize the Youden index (sensitivity + specificity – 1).

Gray zones were calculated using the method defining three levels of response: positive, uncertain, and negative. Uncertain responses were defined using a two-step procedure. We first calculated the 95% CI of the Youden’s index resulting from a 1000 population bootstrap [24]. Then, we determined cut-off values for a sensitivity < 90% or a specificity < 90% (diagnosis tolerance of 10%) [24]. The largest interval from these two steps was used to determine the gray zone [24]. The areas under ROC curves (AUROC) were compared by the Hanley-McNeil test [25]. Correlations were assessed by the Spearman coefficient.

Based on a previous study evaluating the hemodynamic effects of PEEP [26], estimating a difference in the PEEP-test-induced change in CI between responders and non-responders of 0.37 L/min/m^2^, a ratio between responders and non-responders of 1, considering an α risk at 5% and a β risk at 20%, we estimated that the study should include 64 patients. A *p* value < 0.05 was considered significant. The statistical analysis was done using MedCalc 19.2.1 software (MedCalc Software Ltd, Ostend, Belgium).

## Results

### Patient characteristics

The study included 64 patients **(**Additional file 1: Figure S1), whose characteristics are summarized in Table 1. Forty-two (66%) had ARDS, including mild, moderate, and severe ARDS in 14 (33%), 25 (60%), and 3 (7%) patients at the time of evaluation, respectively. The ratio of arterial oxygen partial pressure (PaO_2_) and inspired oxygen fraction was ≤ 150 mmHg in 13 (31%) patients. The etiology of ARDS was COVID-19 in 30 (71%) patients, bacterial pneumonia in seven (17%) patients, pancreatitis in three (7%) patients, and aspiration pneumonia in two (5%) patients.

**Table 1:** Patient characteristics at inclusion

	Total population (*n* = 64)	Volume responsive (*n* = 31)	Volume unresponsive (*n* = 33)	*p* value
*General characteristics*				
Male, *n* (%)	40 (63)	23 (74)	17 (52)	0.106
Age, years	63 ± 15	68 ± 10	58 ± 17	0.005
SAPS II	46 (34–60)	47 (38–63)	40 (33–56)	0.317
Weight, kg	81 (70–95)	76 (67–89)	82 (74–98)	0.087
Height, cm	170 ± 10	170 ± 10	170 ± 10	0.850
ARDS, *n* (%)	42 (66)	20 (65)	22 (67)	0.789
Septic shock, *n* (%)	28 (44)	15 (48)	13 (39)	0.636
Mortality, *n* (%)	26 (41)	15 (48)	11 (33)	0.332
IAP, mmHg	13 (10–15)	13 (11–14)	13 (10–16)	0.644
*Respiratory characteristics*				
Vt, mL/kg predicted body weight	6.2 ± 0.6	6.3 ± 0.7	6.1 ± 0.6	0.494
RR, breaths/min	26 ± 5	26 ± 4	25 ± 5	0.852
PaO_2_/FiO_2_, mmHg	190 (138–266)	185 (135–256)	200 (144–272)	0.722
PEEP, cmH_2_O	12 ± 3	12 ± 2	12 ± 3	0.883
PEEP_t_, cmH_2_O	13 ± 3	13 ± 2	13 ± 3	0.919
Plateau pressure, cmH_2_O	26 ± 4	25 ± 5	27 ± 3	0.090
Driving pressure, cmH_2_O	13 ± 4	12 ± 4	14 ± 3	0.087
Respiratory system compliance, mL/cmH_2_O	34 ± 12	38 ± 15	30 ± 8	0.063
R/I ratio*	0.70 ± 0.29	0.68 ± 0.36	0.72 ± 0.22	0.742
*Hemodynamic characteristics*				
Lactate, mmol/L	1.8 (1.4–2.5)	2 (1.5–3.3)	1.6 (1.3–1.9)	0.009
GEDVi, mL/m^2^	703 ± 188	689 ± 158	715 ± 214	0.215
EVLWi, mL/kg	15 ± 5	14 ± 4	16 ± 6	0.116
PVPI	2.8 (2.2–3.7)	2.7 (2.1–3.7)	2.9 (2.2–3.8)	0.687
Norepinephrine, *n* (%)	57 (89)	27 (87)	30 (91)	0.930
Norepinephrine dosage, µg/kg/min	0.2 (0.1–0.7)	0.3 (0.1–1.1)	0.2 (0.1–0.6)	0.227
Sinus rhythm, *n* (%)	57 (89)	29 (94)	28 (85)	0.475
Atrial extrasystoles, *n* (%)	4 (6)	2 (6)	2 (6)	
Atrial fibrillation, *n* (%)	3 (5)	0	3 (9)	

*ARDS* Acute respiratory distress syndrome, *EVLWi* Extravascular lung water indexed for predicted body weight, *FiO*_*2*_ Fraction of inspired oxygen, *GEDVi* Global end-diastolic volume indexed for body surface, *PaO*_*2*_ Arterial partial pressure of oxygen, *IAP* Intra-abdominal pressure, *PEEP* Positive end-expiratory pressure, *PEEP*_t_ Total positive end-expiratory pressure, *PVPI* Pulmonary vascular permeability index, *R/I* Recruitment-to inflation, *RR* Respiratory rate, *SAPS II* Simplified acute physiology score II, *Vt* Tidal volume

Values are expressed as *n* (%), mean ± standard deviation or median (interquartile range)

The *p* value corresponds to the comparison between volume responders and volume non-responders

^*^Calculated in 20 volume responsive patients and 22 volume non-responsive patients

Acute circulatory failure was attributed to septic shock in 28 (44%) patients, vasoplegic non-septic shock in 33 (52%) patients, and cardiogenic shock in three (5%) patients. Inclusion occurred one (1–3) day after shock onset. No patient had spontaneous breathing at the time of inclusion, and neuromuscular blockade was used in 33 (52%) patients. All patients were in the supine position and no patient presented *acute cor pulmonale* [27]. Measurements of IAP were available in 56 (88%) patients. The IAP was 13 (10–15) mmHg.

In ARDS patients, the R/I ratio was 0.75 (0.53–0.86). In patients with an R/I ratio lower than the median, defined as “low recruiters”, its value was 0.49 (0.38–0.58). In patients with an R/I ratio above the median, defined as “high recruiters”, its value was 0.86 (0.81–1.01). The airway opening pressure in these 42 patients was 0 (0–5) cmH_2_O.

### Hemodynamic effects of passive leg raising

Hemodynamic variables at baseline are reported in Tables 1 and 2. During PLR, CI increased by 14% (11–16%) in 31 (48%) volume responsive patients and by 2% (1–5%) in volume unresponsive patients (*p* < 0.0001).

**Table 2:** Hemodynamic and respiratory variables during the study protocol

	Baseline 1 (n = 64)	PLR (n = 64)	Baseline 2 (n = 64)	PEEP-test (n = 64)	Baseline 3 (n = 64)	Volume expansion (n = 30)
*PEEP, mmHg*						
Volume responsive Volume unresponsive	13 ± 2 12 ± 3	13 ± 2 12 ± 3	13 ± 2 12 ± 3	5 ± 0 5 ± 0	13 ± 2 12 ± 3	13 ± 2
*RR, /min*						
Volume responsive Volume unresponsive	26 ± 4 26 ± 5	26 ± 4 26 ± 5	26 ± 4 26 ± 5	26 ± 4 26 ± 5	26 ± 4 26 ± 5	26 ± 4
*SpO*_2_*, %*						
Volume responsive Volume unresponsive	94 ± 2 94 ± 3	94 ± 3 94 ± 3	94 ± 2 94 ± 3	93 ± 4^¤^ 93 ± 4^¤^	94 ± 3 94 ± 3	96 ± 2^1^
*HR, beats/min*						
Volume responsive Volume unresponsive	84 ± 21 78 ± 23	83 ± 22 78 ± 22	85 ± 23 79 ± 24	85 ± 23 78 ± 23	85 ± 21 78 ± 23	78 ± 20^1^
*SAP, mmHg*						
Volume responsive Volume unresponsive	120 ± 25 120 ± 25	134 ± 27* 128 ± 21*	120 ± 25 119 ± 21	130 ± 26^¤^ 125 ± 20^¤^	120 ± 26 120 ± 21	138 ± 35^1^
*DAP, mmHg*						
Volume responsive Volume unresponsive	57 ± 13 57 ± 15	62 ± 13* 60 ± 14*	57 ± 12 56 ± 14	59 ± 12^¤^ 57 ± 14^¤^	56 ± 12 56 ± 14	60 ± 14^1^
*MAP, mmHg*						
Volume responsive Volume unresponsive	77 ± 16 78 ± 16	87 ± 18* 83 ± 15*	77 ± 16 76 ± 15	83 ± 18^¤^ 80 ± 15^¤^	77 ± 17 77 ± 14	87 ± 22^1^
*PP, mmHg*						
Volume responsive Volume unresponsive	61 ± 23 63 ± 23	72 ± 25* 68 ± 19*	63 ± 22 63 ± 18	71 ± 22^¤^ 66 ± 22	64 ± 22 64 ± 18	77 ± 29 ±
*CVP, mmHg*						
Volume responsive Volume unresponsive	9 ± 3 10 ± 4	12 ± 3* 14 ± 4*	8 ± 3 10 ± 4	7 ± 3^¤^ 8 ± 4^¤^	8 ± 3 10 ± 4	11 ± 3^1^
*CI, L/min/m*^2^						
Volume responsive Volume unresponsive	2.4 ± 0.7 2.7 ± 0.7	2.8 ± 0.9* 2.8 ± 0.7	2.4 ± 0.7 2.7 ± 0.7	2.9 ± 0.9^¤^ 2.9 ± 0.7^¤^	2.4 ± 0.7 2.7 ± 0.7	2.8 ± 0.7^1^
*PPV, %*						
Volume responsive Volume unresponsive	9 ± 6 7 ± 6	7 ± 6 6 ± 5	10 ± 6 6 ± 4^$^	6 ± 5^¤^ 6 ± 4	9 ± 6 6 ± 5	6 ± 5^1^
*SVV, %*						
Volume responsive Volume unresponsive	10 ± 6 8 ± 5	7 ± 6* 6 ± 4*	11 ± 6 6 ± 4^$^	8 ± 6^¤^ 7 ± 5	10 ± 7 7 ± 5^$^	7 ± 6^1^

^*^p < 0.05 PLR *vs.* Baseline 1

^¤^ *p* < 0.05 PEEP-test *vs.* Baseline 2

^1^ *p* < 0.05 Volume expansion *vs.* Baseline 3

^$^ *p* < 0.05 responders *vs.* non-responders

*CVP* Central venous pressure, *CI* Cardiac index, *DAP* Diastolic arterial pressure, *HR* Heart rate, *MAP* Mean arterial pressure, *PEEP* Positive end-expiratory pressure *PLR* Passive leg raising, *PPV* Pulse pressure variation, *RR* Respiratory rate, *SAP* Systolic arterial pressure, *SVV* Stroke volume variation

Values are expressed as mean ± standard deviation

### Respiratory and hemodynamic effects of the PEEP-test

The PEEP-test consisted of a 7 (5–10) cmH_2_O decrease in PEEP (Table 2). During the test, SpO_2_ decreased by 1 (0–3)% in absolute value from 94 ± 3% (*p* < 0.001), with no difference between volume responders and non-responders (Table 2). In the 23 (36%) patients in whom SpO_2_ decreased ≥ 2%, the time to resaturation after the PEEP-test was 50 (34–109) seconds. During the PEEP-test, desaturation to an SpO_2_ < 90% occurred in six (9%) patients, the lowest value being 82%, reached by one patient. After stabilization at Baseline 3, SpO_2_ was ≥ 90% in all patients.

During the PEEP-test, heart rate changed neither in volume responders nor in non-volume responders. On the contrary, arterial pressure increased, regardless of the volume responsiveness status (Table 2). The PEEP-test increased CI from 2.6 ± 0.7 to 2.9 ± 0.8 L/min/m^2^. The maximum value of CI during the one-minute PEEP-test was reached within 37 ± 14 s. There was no correlation between the amplitude of the PEEP change and the PEEP-test-induced increase in CI (*r* = 0.097 (− 0.150–0.336; *p* = 0.438)).

**Fig. 1:**
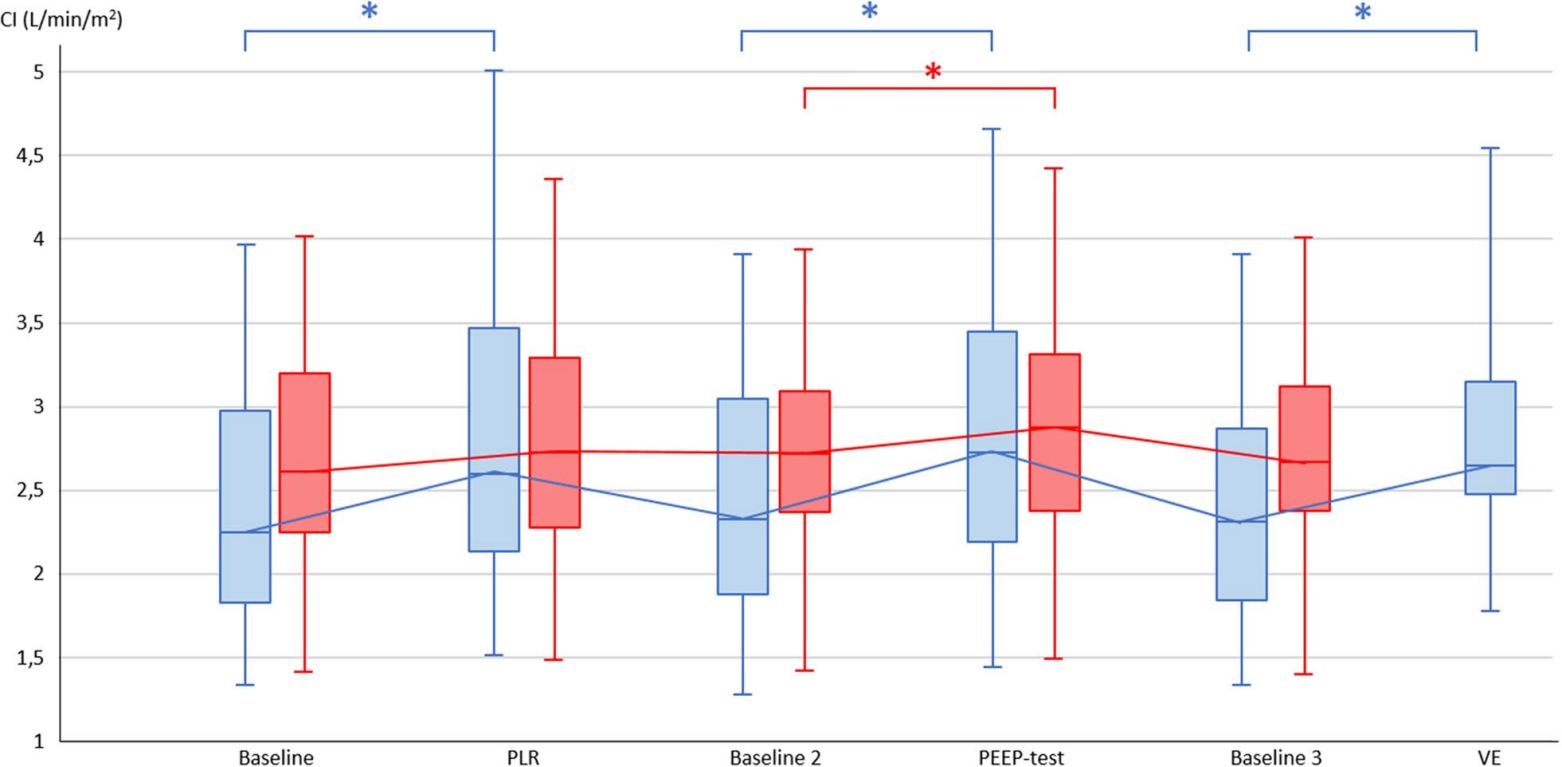
Changes in cardiac index with passive leg raising, PEEP-test and volume expansion in volume responsive and volume unresponsive patients. *CI* Cardiac index, *PEEP* Positive end-expiratory pressure, *PLR* Passive leg raising, *VE* Volume expansion. Volume responsive patients are represented in blue and volume unresponsive patients in red. Volume expansion was performed in 30 volume responsive patients. *p < 0.05

The PEEP-test increased CI to a larger extent in volume responders than in non-responders, in absolute value (Table 2**, **Figs. 1**,** Additional file 1: S2 and S3) as in percentage (3% (1–7%) *vs.* 13% (9–17%), respectively, *p* < 0.0001). The PEEP-test increased PP to a larger extent in volume responders than in non-responders, in absolute value (Table 2) as in percentage (12% (7–21%) *vs.* 4% (1–11%), respectively, *p* < 0.0001). PPV and SVV significantly decreased in volume responders and remained unchanged in volume non-responders (Table 2). In patients in whom the R/I ratio was measured, the hemodynamic response to the PEEP-test was similar in patients in whom the R/I ratio was ≥ 0.75 and their counterparts (Additional file 1: Table S1).

### Ability of the PEEP-test to detect volume responsiveness

**Fig. 2:**
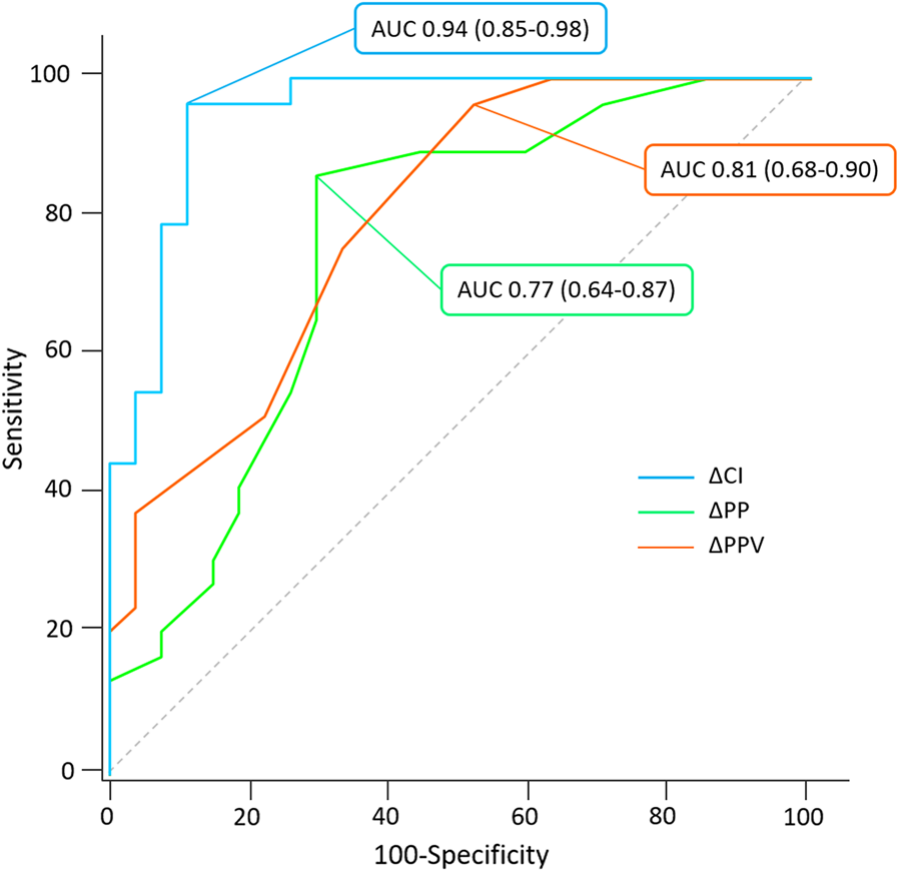
Area under the receiver-operating characteristic curves expressing the ability to detect volume responsiveness of changes in cardiac index, absolute changes in pulse pressure variation and changes in pulse pressure during a PEEP-test. *AUC* Area under the receiver-operating characteristic curve, *CI* Cardiac index, *PP* Pulse pressure, *PPV* Pulse pressure variation

**Fig. 3:**
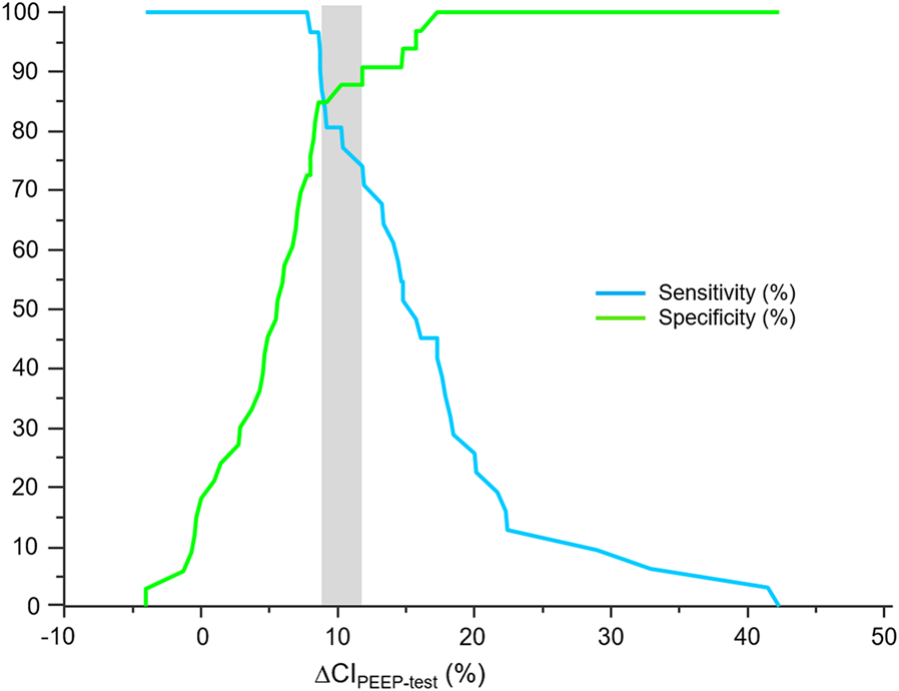
Sensitivity and specificity of the changes in cardiac index induced by the PEEP-test depending on the test result. The gray zone represents the uncertain zone with cut-off values with a sensitivity of < 90% or a specificity of < 90%. ∆CI_PEEP-test_: changes in cardiac index induced by the decrease in positive end-expiratory pressure from baseline to 5 cmH_2_O

A PEEP-test-induced increase in CI > 8.6% predicted a positive PLR-test with a sensitivity of 96.8% (95%CI 83.3–99.9%) and a specificity of 84.9% (95%CI 68.1–94.9%) (Additional file 1: Table S2). The AUROC was 0.94 (0.85–0.98) (*p* < 0.0001 *vs.* 0.5) **(**Fig. 2**)**. The likelihood ratio for this threshold was 6.4 (2.8–14.4), with a positive predictive value of 85.7% (69.7–95.2%) and a negative predictive value of 96.6% (82.2–99.9%). The gray zone ranged between 8.7% and 11.8%, in which six (9%) patients were situated (Fig. 3). The correlation between the increases in CI during the PEEP- and PLR-tests was significant (*r* = 0.76 (0.63–0.85), *p* < 0.0001) (Additional file 1: Figure S4).

The AUROC for the PEEP-test-induced changes in stroke volume was 0.93 (0.84–0.98, *p* < 0.0001 *vs.* 0.5) (Additional file 1: Figure S5), which was not different from the AUROC of the PEEP-induced changes in CI (*p* = 0.8733). If considering the PEEP-test-induced changes in stroke volume, one single patient became true negative while being a false negative when considering changes in CI. The correlation between the increases in stroke volume during PEEP- and PLR-tests was significant (*r* = 0.71 (0.57–0.81), *p* < 0.0001) (Additional file 1: Figure S6), and not different from the previous one (*p* = 0.5934).

In the 57 patients with no cardiac arrhythmia, a decrease of PPV ≥ 1% (in absolute value) during the PEEP-test detected volume responsiveness with an AUROC of 0.81 (95%CI 0.68–0.90) (*p* < 0.0001 *vs.* 0.5), a sensitivity of 96.6% (95%CI 82.2%-99.9%) and a specificity of 50.0% (95%CI 30.6–69.4%) (Figs. 2 and Additional file 1: Figure S7). This AUROC was significantly lower than for the PEEP-test-induced changes in CI (*p* = 0.023) (Fig. 2). In this subgroup of patients, an increase in PP ≥ 4 mmHg during the PEEP-test detected volume responsiveness with an AUROC of 0.75 (95%CI 0.62–0.85), with a sensitivity of 80.7% (62.5–92.5%) and a specificity of 69.7% (95%CI 51.3–84.4%) (Figs. 2 and Additional file 1: Figure S8). This AUROC was lower than for the PEEP-test-induced changes in CI (*p* = 0.012), but not different than for the PEEP-test-induced changes in PPV (*p* = 0.602) (Fig. 2).

In patients in whom the R/I ratio was determined, the AUROC was similar in high and low recruiters (0.97 (0.82–1.00) *vs.* 0.94 (0.65–1.00), respectively, p = 0.7880) (Additional file 1: Figure S9).

### Volume expansion

Volume expansion was performed in 30 patients with a positive PLR-test. The CI increased by 18% (16–30%) on average (Table 2). In one patient, the CI measured by transpulmonary thermodilution increased by < 15% with fluid infusion. In this patient, the fluid-induced increase in CI was 9%, whereas the PLR-induced increase in CI was 11% and the PEEP-test-induced increase in CI was 22%.

## Discussion

In patients receiving invasive mechanical ventilation with a PEEP ≥ 10 cmH_2_O, an increase in CI larger than 8.6% during a PEEP-test, which consists of a transient decrease in PEEP to 5 cmH_2_O, reliably detects volume responsiveness. The changes in CI induced by the PEEP-test perform better than the changes in arterial PP and PPV.

Fluids should be considered as drugs with inconsistent efficacy and several adverse effects [28, 29]. Thus, unnecessary fluid infusion should be avoided [30, 31] and several tests and indices are today available to assess volume unresponsiveness [3]. Some of them do so by taking advantage of heart–lung interactions in patients under invasive mechanical ventilation. Using PPV and SVV is limited by the numerous conditions of validity that must be fulfilled [5]. The end-expiratory occlusion test is well established [32], but it requires that patients tolerate a 15-s occlusion of the ventilator, which is sometimes impossible without deep sedation [12]. The tidal volume challenge is easy to perform, but is less validated, and the diagnostic threshold reported by validation studies varies [3, 33].

Changing PEEP may be another way to test volume responsiveness. PEEP is transmitted to the intrathoracic pressure, which surrounds the cardiac chambers, and to the right atrium. By decreasing PEEP, the PEEP-test reduces the intramural right atrial pressure (RAP), which is the downstream pressure of venous return [34]. As only a part of the decrease in alveolar pressure is transmitted to the right atrium, the intrathoracic pressure should decrease more than the intramural RAP, so that the transmural RAP should increase. Our results suggest that this preload challenge was of sufficient amplitude to assess volume responsiveness. The PEEP-test assessed on CI detected volume responders with a large AUROC, and few patients in the gray zone. As heart rate was roughly unchanged by the PEEP-test, a similar reliability was obtained with the PEEP-test-induced changes in stroke volume.

Besides changing cardiac preload, PEEP also affects right ventricular afterload by increasing transpulmonary pressure [13, 26]. This effect is independent from preload responsiveness. Accordingly, in volume non-responders, this phenomenon explained the significant increase in CI we observed during the PEEP test. In volume responders, the larger increase in CI during the test may have resulted from the sum of both effects, the decrease in right ventricular afterload, and the increase in cardiac preload. Of note, we did not include patients with *acute cor pulmonale*, in which the PEEP-test-induced decrease in right ventricular afterload could be so strong that it would largely increase CI even in case of volume responsiveness. This is a limitation of our study.

Our results may help understanding the hemodynamic effects of PEEP. It has been suggested that the effect of PEEP on cardiac preload is minimal because of two phenomena. First, PEEP is transmitted to the abdominal cavity, increasing the upstream pressure of venous return, attenuating the effect on its pressure gradient [35, 36]. Second, the PEEP-induced augmentation in right ventricular afterload may exacerbate the increase in intramural right atrial pressure, so that it may increase to the same extent as the intrathoracic pressure. Accordingly, some studies observed that the transmural RAP remained unchanged in some patients when PEEP was increased [37, 38]. Our results rather suggest that the effects of PEEP on cardiac preload are not negligible, otherwise we would have observed a large proportion of false negatives to the PEEP-test. Nevertheless, we neither measured the pleural pressure nor estimated the mean systemic pressure, so that we can only make assumptions on this issue.

Another phenomenon may have induced some false negatives to the PEEP-test. In theory, reducing PEEP may not decrease, but conversely increase right ventricular afterload, because of lung derecruitment and/or hypoxic pulmonary vasoconstriction. This may counterbalance the increase in CI due to volume responsiveness in volume responders. However, this theoretical phenomenon was unlikely. First, hypoxic pulmonary vasoconstriction takes a couple of minutes to occur, so that it was unlikely during the 1-min PEEP-test [39, 40]. Second, in the only false negative to the PEEP-test, central venous pressure decreased during the test, while it would have increased in the case of marked increase in right ventricular afterload. Regarding the issue of lung derecruitment during the PEEP-test, it may increase pulmonary vascular resistance if the lung volume decreases below the functional residual capacity, as a result of the U shape of the relationship between pulmonary vascular resistance and lung volume [41]. We may have included a specific population of high lung recruiters, as the R/I ratio in ARDS patients was rather high compared to previous studies [19, 42], so that this phenomenon may have been significant. However, the AUROC of the PEEP-test was identical in high and low lung-recruiters, at least as classified through the R/I ratio in ARDS patients. As we did not specifically investigate this hypothesis, e.g., by assessing the size of the right cavities by echocardiography, we cannot strictly exclude it. Nevertheless, this phenomenon may have induced a large number of false negatives, which we did not observe.

Of note, the effects of PEEP, and thus of the PEEP-test, may depend on the volume status [43]. A low central blood volume favors the extent of West zones 2 conditions [26]. Decreasing PEEP may increase the central blood volume and reduce the pulmonary vascular resistance through this mechanism. This may have contributed to the increase in CI observed in volume responsive patients during the test. Note that fluid administration or PLR also increase central blood volume and may decrease pulmonary vascular resistance in the same way. Accordingly, we used the term “volume” rather than “preload” responsiveness in the present study.

A study in critically ill patients showed that the response of stroke volume to a transient increase in PEEP by 5 cmH_2_O also predicted fluid responsiveness [16]. Our results confirm that volume responsiveness can be assessed by manipulating PEEP. The latter study was conducted mainly in post-operative patients, with lower PEEP level at baseline (6 cmH_2_O) than in our study in patients with circulatory failure (13 ± 3 cmH_2_O). Our PEEP-test, consisting in decreasing PEEP, is more convenient in such patients with higher PEEP at baseline.

We observed that the reliability of the PEEP-test when assessed through changes in PP was low, confirming a previous study [44]. Conversely, the PEEP-test kept its reliability when observing changes in PPV, although it was significantly lower than for changes in CI. Note that this result was obtained when excluding patients with atrial fibrillation. Nevertheless, in patients with sinus rhythm, this positive result suggests that, like the tidal volume challenge [45], the PEEP-test may be a convenient way to assess volume responsiveness in patients with an arterial catheter and no cardiac output monitoring.

Considering all patients, the PEEP-test significantly decreased SpO_2,_ regardless of the volume responsiveness status. This desaturation was limited and rapidly reversible, certainly because of the short duration of the PEEP decrease. Even though the lowest decrease of SpO_2_ during the PEEP-test in our study remained in the safe range, the test should be interrupted in case of deep desaturation.

Beyond those mentioned above, our study has several limitations. First, not all patients received fluid administration, because we thought it was unethical to administer a fluid bolus even in the absence of volume responsiveness in critically ill patients, including some with ARDS, in whom an increased fluid balance is an independent risk factor of mortality [46]. Nevertheless, PLR predicts fluid responsiveness very reliably [9, 11]. Accordingly, among the patients with a positive PLR and who received fluid, all but one was fluid responsive. Second, we included only intubated patients, while the PEEP-test may also be used in patients with non-invasive ventilation. Third, we included patients with PEEP at baseline ≥ 10 cmH_2_O, so that the reliability of this test is unclear in patients with lower PEEP. Fourth, some of our patients had a higher IAP, while some studies suggest this condition may induce some false negatives to the PLR-test [47, 48]. However, this hypertension was mild in our population. Finally, we did not assess volume responsiveness when PEEP was 5 cmH_2_O, although some patients may have been volume responsive at the high PEEP but volume unresponsive when PEEP was decreased.

## Conclusion

In critically ill patients on mechanical ventilation with PEEP ≥ 10 cmH_2_O and no spontaneous ventilation, volume responsiveness can be reliably evaluated by an increase in CI higher than 8.6% during a PEEP-test, which consists in decreasing PEEP to 5 cmH_2_O. In patients with sinus rhythm, volume responsiveness can also be reliably evaluated by a decrease in PPV (1% in absolute value) during a PEEP-test, although the diagnostic ability is lower than for PEEP-test-induced changes in CI.

## Supplementary Information

**Additional file 1**. Supplementary tables and figures.

## Data Availability

The datasets used and/or analyzed during the current study are available from the corresponding author on reasonable request.

## Abbreviations

AOP: Airway opening pressure
ARDS: Acute respiratory distress syndrome
AUC: Area under ROC curves
CI: Cardiac index
PaO_2_: Arterial oxygen partial pressure
PEEP: Positive end-expiratory pressure
PP: Pulse pressure
PPV: Pulse pressure variation
PLR: Passive leg raising
R/I: Recruitment-to-inflation
RAP: Right atrial pressure
ROC: Receiver-operating characteristic
SpO_2_: Pulse oxygen saturation
SVV: Stroke volume variation
∆CI: Percent change in cardiac index
∆PP: Percent change in arterial pulse pressure
∆PPV: Percent change in PPV
