# Supplementary material for: The increase in cardiac output induced by a decrease in positive end-expiratory pressure reliably detects volume responsiveness: the PEEP-test study
Source: Crit Care. 2023 Apr 9;27:136. doi: 10.1186/s13054-023-04424-7 (PMC10082988; doi:10.1186/s13054-023-04424-7)
Supplement: Supplementary file 1 — Additional file 1. Supplementary tables and figures. [file 13054_2023_4424_MOESM1_ESM.docx]

**Online supplementary material**

**The increase in cardiac output induced by a decrease in positive end-expiratory pressure reliably detects preload responsiveness: the PEEP-test study**

Christopher LAI, Rui SHI, Alexandra BEURTON, Francesca MORETTO, Soufia AYED, Nicolas FAGE, Francesco GAVELLI, Arthur PAVOT, Martin DRES, Jean-Louis TEBOUL, Xavier MONNET.

**Table of contents**

[Appendix 1. STARD checklist 2](#_Toc129181510)

[Figure S1. Study flow-chart 4](#_Toc129181511)

[Figure S2. Individual changes in cardiac index during the PEEP-test in absolute value 5](#_Toc129181512)

[Figure S3. Changes in cardiac index during PEEP-test in percentage 6](#_Toc129181513)

[Figure S4. Correlation between changes in cardiac index during passive leg raising and the PEEP-test 7](#_Toc129181514)

[Figure S5. Performance of changes in stroke volume index during the PEEP-test to detect volume responsiveness 8](#_Toc129181515)

[Figure S6. Correlation between changes in stroke volume index during passive leg raising and the PEEP-test 9](#_Toc129181516)

[Figure S7. Performance of changes in pulse pressure variation (expressed in absolute value) during the PEEP-test to detect volume responsiveness in patients with no atrial fibrillation (n=57) 10](#_Toc129181517)

[Figure S8. Performance of changes in pulse pressure during the PEEP-test to detect volume responsiveness in patients with no atrial fibrillation (n=57) 11](#_Toc129181518)

[Figure S9. Receiver-operating characteristic curves expressing the performance of changes in cardiac index during the PEEP-test to detect volume responsiveness in low-recruiters (n=21) and high-recruiters (n=21) patients 12](#_Toc129181519)

[Table S1. Hemodynamic variables during the study protocol in high recruiters and low recruiters (n=42) 13](#_Toc129181520)

[Table S2. Variability of diagnostic accuracy of changes in cardiac index during PEEP-test to detect volume responsiveness 15](#_Toc129181521)

# Appendix 1. STARD checklist

|  | **Section & Topic** | **No** | **Item** | **Reported on page #** |
| --- | --- | --- | --- | --- |
|  |  |  |  |  |
|  | **TITLE OR ABSTRACT** |  |  |  |
|  |  | **1** | Identification as a study of diagnostic accuracy using at least one measure of accuracy  (such as sensitivity, specificity, predictive values, or AUC) | Page 1, lines 1-3 |
|  | **ABSTRACT** |  |  |  |
|  |  | **2** | Structured summary of study design, methods, results, and conclusions  (for specific guidance, see STARD for Abstracts) | Page 2, 3 |
|  | **INTRODUCTION** |  |  |  |
|  |  | **3** | Scientific and clinical background, including the intended use and clinical role of the index test | Page 4, lines 62-83 |
|  |  | **4** | Study objectives and hypotheses | Page 4, lines 84-87 |
|  | **METHODS** |  |  |  |
|  | *Study design* | **5** | Whether data collection was planned before the index test and reference standard  were performed (prospective study) or after (retrospective study) | Page 5, line 91 |
|  | *Participants* | **6** | Eligibility criteria | Page 5, lines 97-107 |
|  |  | **7** | On what basis potentially eligible participants were identified  (such as symptoms, results from previous tests, inclusion in registry) | Page 6, lines 111-112 |
|  |  | **8** | Where and when potentially eligible participants were identified (setting, location and dates) | Page 5, lines 100-101 |
|  |  | **9** | Whether participants formed a consecutive, random or convenience series | Page 5, lines 107 |
|  | *Test methods* | **10a** | Index test, in sufficient detail to allow replication | Page 7, lines 146-150 |
|  |  | **10b** | Reference standard, in sufficient detail to allow replication | Page 7, lines 142-145 |
|  |  | **11** | Rationale for choosing the reference standard (if alternatives exist) | Page 4, line 64-69 |
|  |  | **12a** | Definition of and rationale for test positivity cut-offs or result categories  of the index test, distinguishing pre-specified from exploratory | Page 8, lines 164-168 |
|  |  | **12b** | Definition of and rationale for test positivity cut-offs or result categories  of the reference standard, distinguishing pre-specified from exploratory | Page 7, lines 142-143 |
|  |  | **13a** | Whether clinical information and reference standard results were available  to the performers/readers of the index test | Page 6, line 138-150 |
|  |  | **13b** | Whether clinical information and index test results were available  to the assessors of the reference standard | Page 7, line 138-150 |
|  | *Analysis* | **14** | Methods for estimating or comparing measures of diagnostic accuracy | Page 8, lines 174-175 |
|  |  | **15** | How indeterminate index test or reference standard results were handled | NA |
|  |  | **16** | How missing data on the index test and reference standard were handled | NA |
|  |  | **17** | Any analyses of variability in diagnostic accuracy, distinguishing pre-specified from exploratory | Page 8, lines 164-175 |
|  |  | **18** | Intended sample size and how it was determined | Page 8, lines 176-179 |
|  | **RESULTS** |  |  |  |
|  | *Participants* | **19** | Flow of participants, using a diagram | Figure S1 |
|  |  | **20** | Baseline demographic and clinical characteristics of participants | Page 9, lines 184-201, table 1 |
|  |  | **21a** | Distribution of severity of disease in those with the target condition | Page 9, lines 186-193 and 198-201, table 1 |
|  |  | **21b** | Distribution of alternative diagnoses in those without the target condition | NA |
|  |  | **22** | Time interval and any clinical interventions between index test and reference standard | Page 7, line 146 |
|  | *Test results* | **23** | Cross tabulation of the index test results (or their distribution)  by the results of the reference standard | Figure S2 |
|  |  | **24** | Estimates of diagnostic accuracy and their precision (such as 95% confidence intervals) | Page 11, lines 232-237 |
|  |  | **25** | Any adverse events from performing the index test or the reference standard | Page 10, line 213-215 |
|  | **DISCUSSION** |  |  |  |
|  |  | **26** | Study limitations, including sources of potential bias, statistical uncertainty, and generalisability | Page 16, lines 356-369 |
|  |  | **27** | Implications for practice, including the intended use and clinical role of the index test | Page 12, lines 268-271 |
|  | **OTHER INFORMATION** |  |  |  |
|  |  | **28** | Registration number and name of registry | Page 18, line 401 |
|  |  | **29** | Where the full study protocol can be accessed | Page 18, line 413 |
|  |  | **30** | Sources of funding and other support; role of funders | Page 18, line 423 |
|  |  |  |  |  |

# Figure S1. Study flow-chart


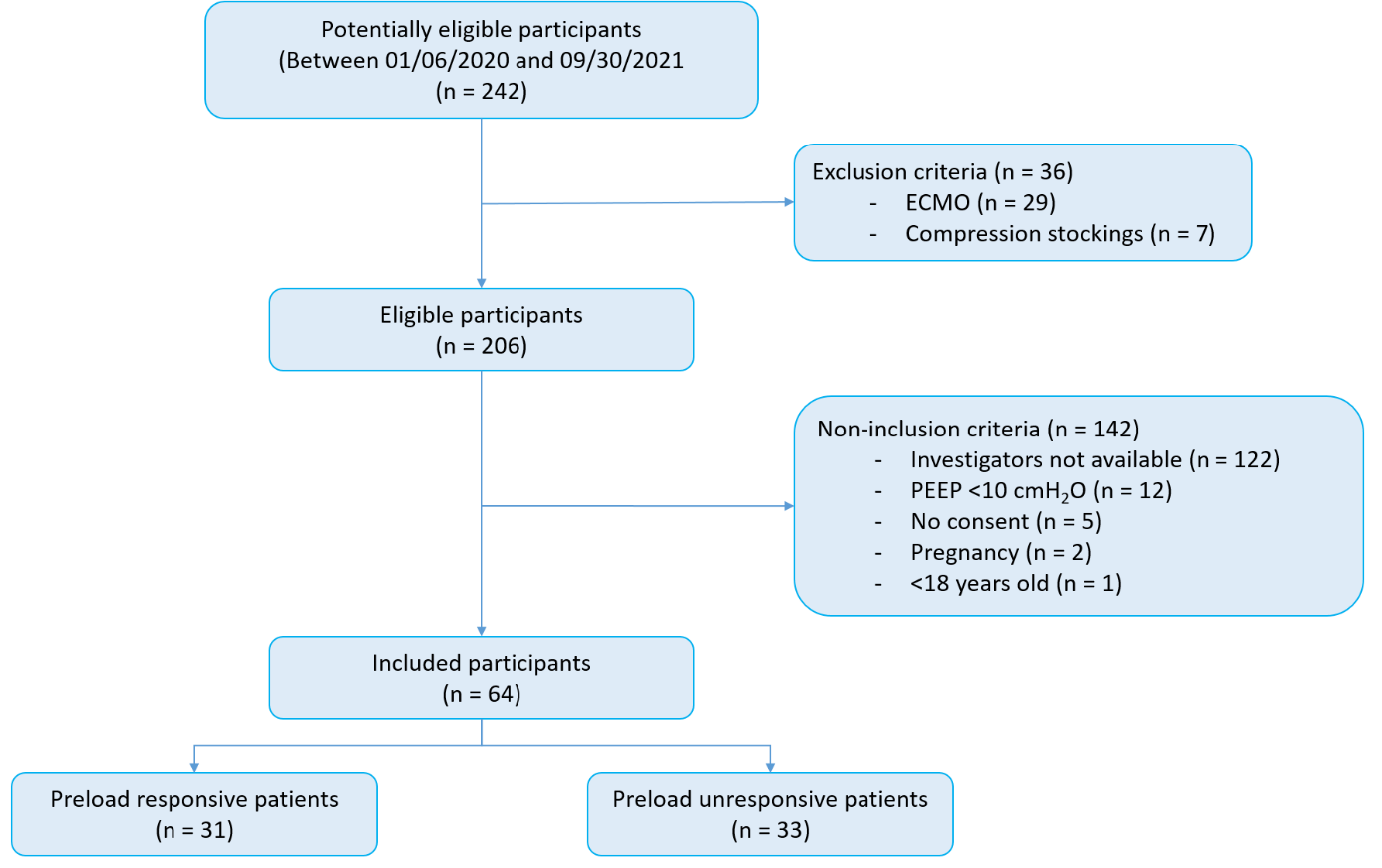


ECMO : extracorporeal membrane oxygenation ; PEEP : positive end-expiratory pressure

# Figure S2. Individual changes in cardiac index during the PEEP-test in absolute value


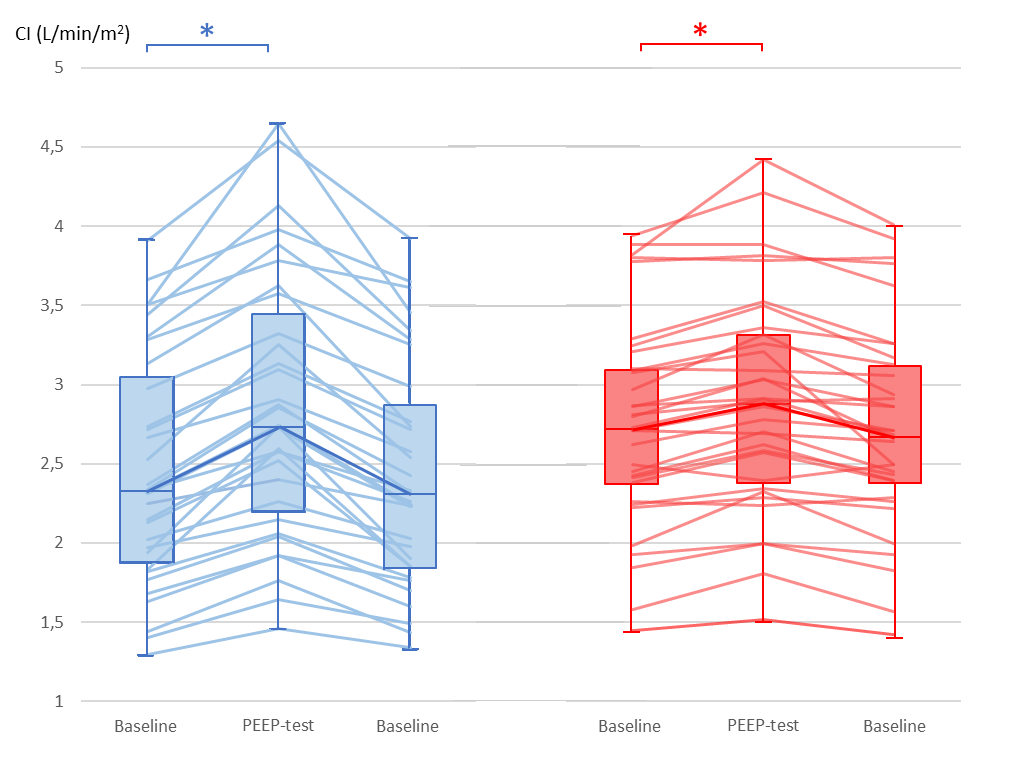


CI: cardiac index; PEEP: positive end-expiratory pressure

Blue: volume responsive patients; Red: volume unresponsive patients

* p<0.05 vs. Baseline

# Figure S3. Changes in cardiac index during PEEP-test in percentage


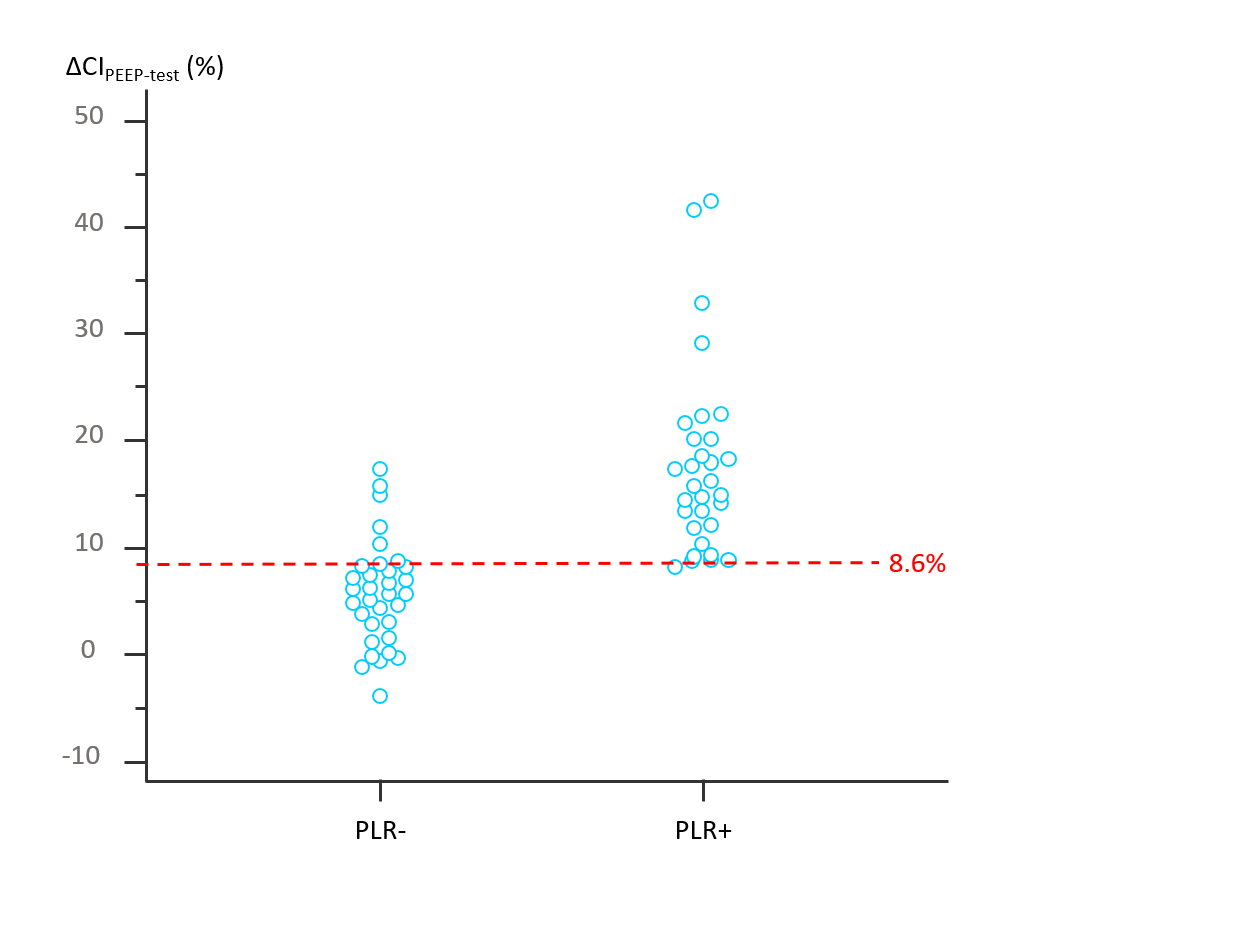


∆CI: changes in cardiac index; PEEP: positive end-expiratory pressure; PLR: passive leg raising

# Figure S4. Correlation between changes in cardiac index during passive leg raising and the PEEP-test


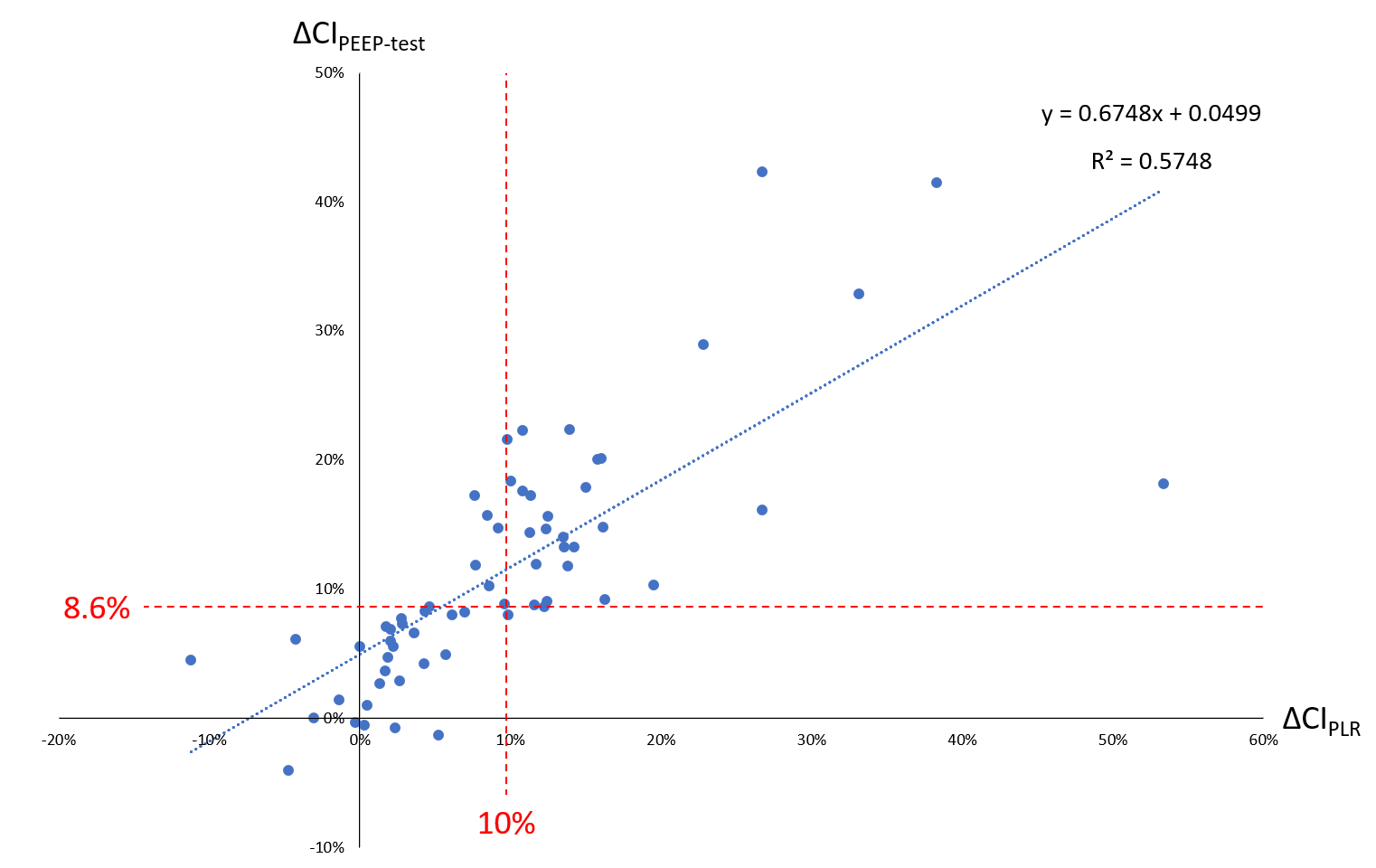


∆CI: changes in cardiac index; PEEP: positive end-expiratory pressure; PLR: passive leg raising

# Figure S5. Performance of changes in stroke volume index during the PEEP-test to detect volume responsiveness

**
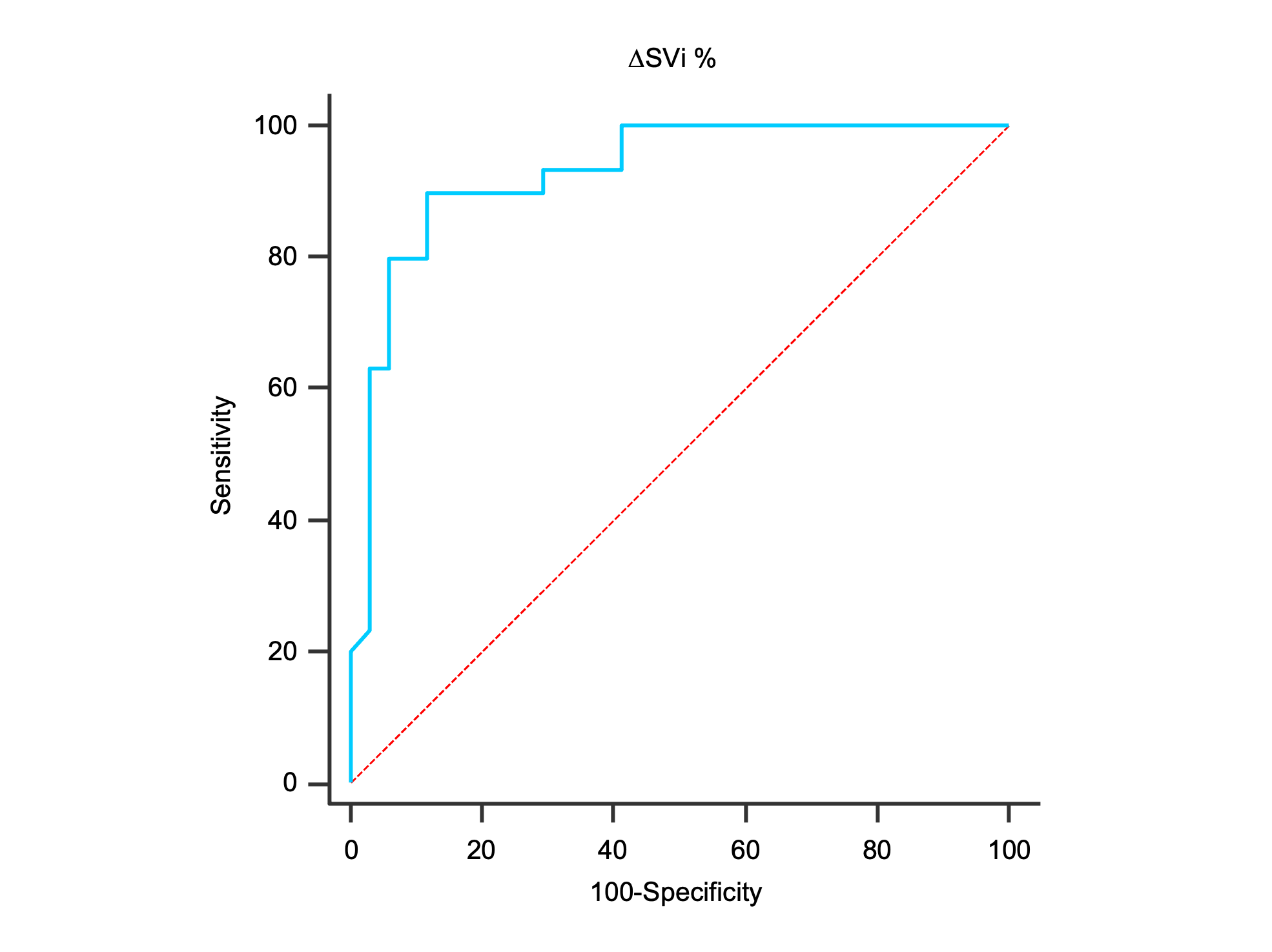
**

**
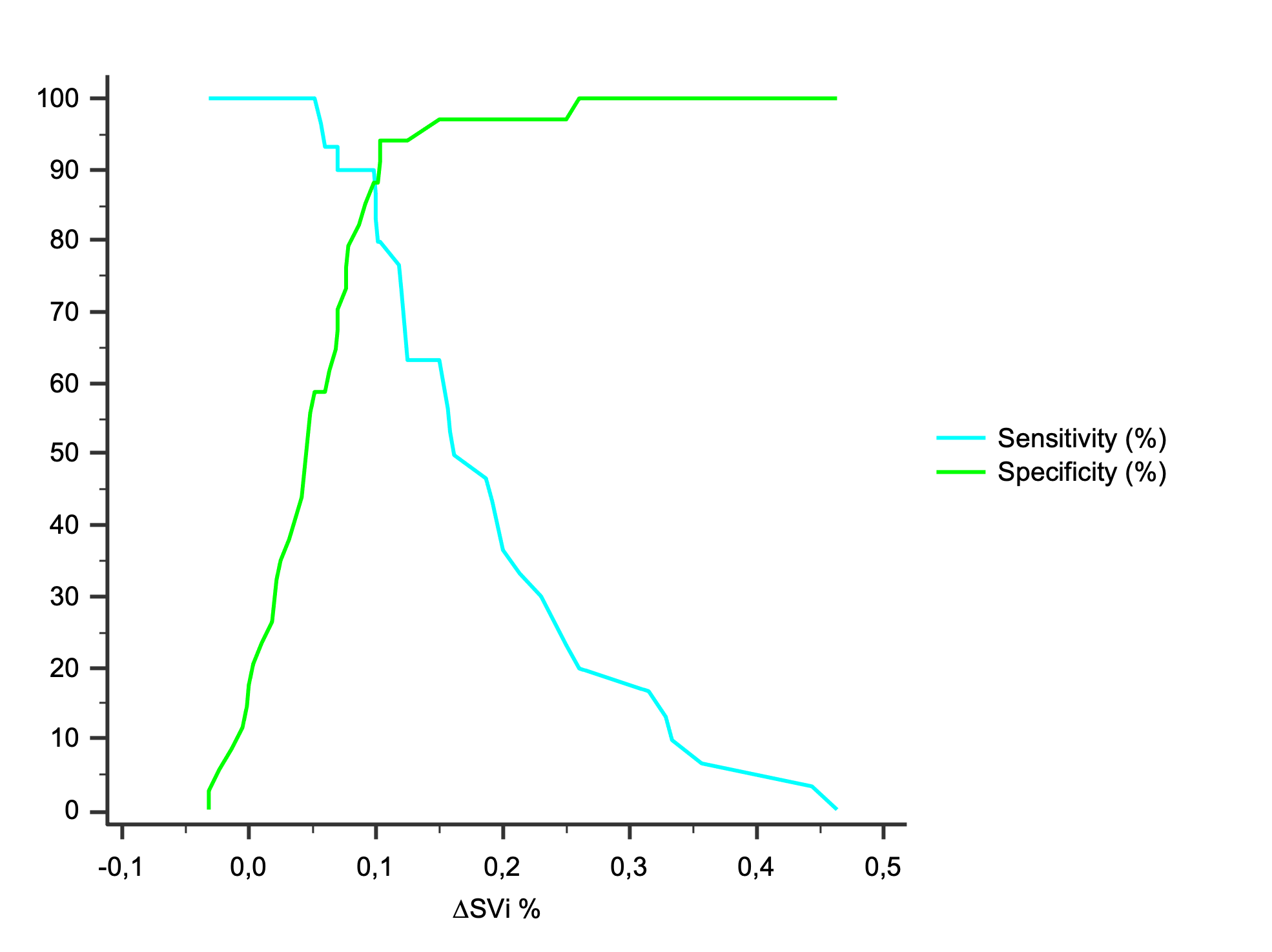
**

**Upper panel:** Receiver-operating characteristic curves expressing the ability of changes in stroke volume index during the PEEP-test to detect volume responsiveness.

**Bottom panel:** Sensitivity and specificity of the changes in stroke volume index induced by the PEEP-test depending on the test result.

∆SVi: change in stroke volume index; PEEP: positive end-expiratory pressure

# Figure S6. Correlation between changes in stroke volume index during passive leg raising and the PEEP-test


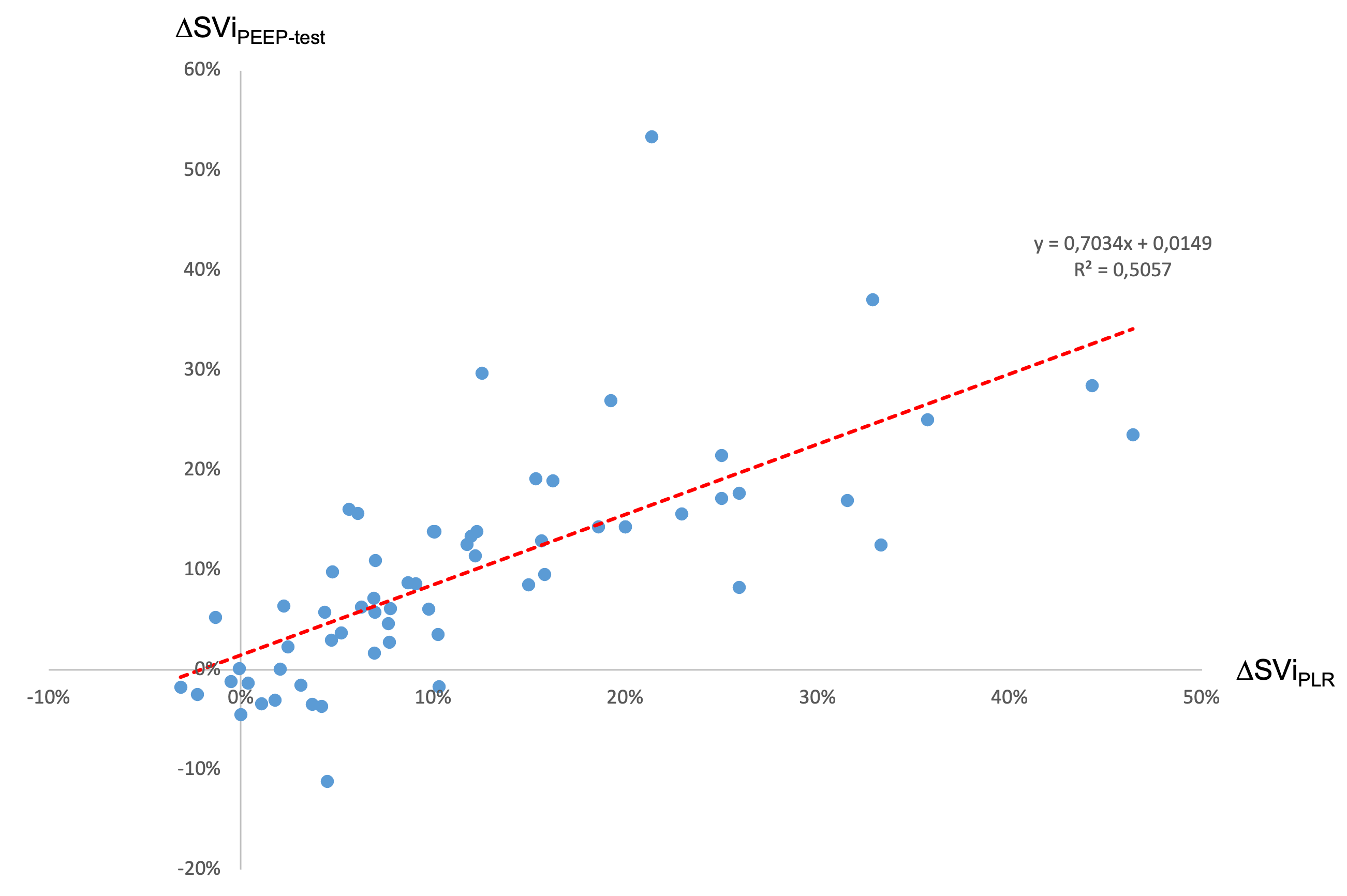


∆SVI: changes in stroke volume index; PEEP: positive end-expiratory pressure; PLR: passive leg raising

# Figure S7. Performance of changes in pulse pressure variation (expressed in absolute value) during the PEEP-test to detect volume responsiveness in patients with no atrial fibrillation (n=57)


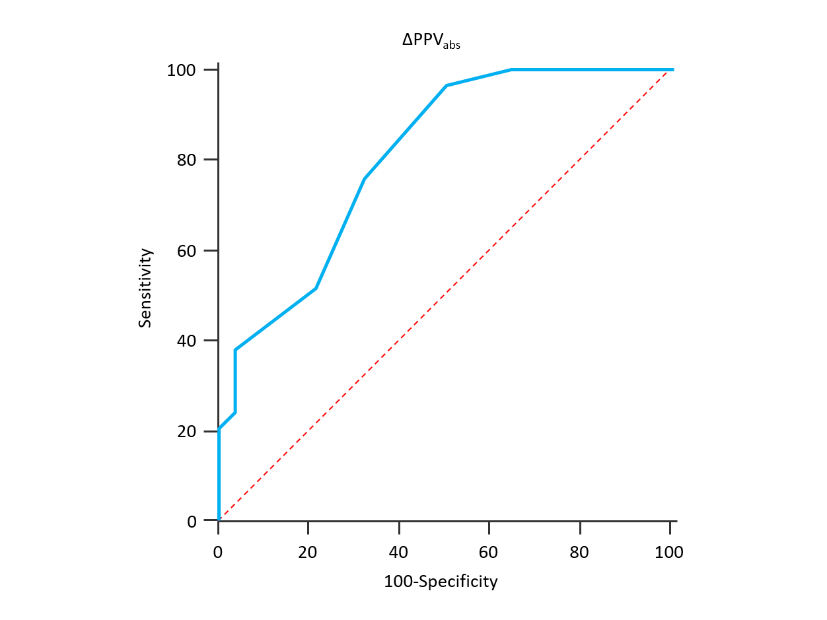


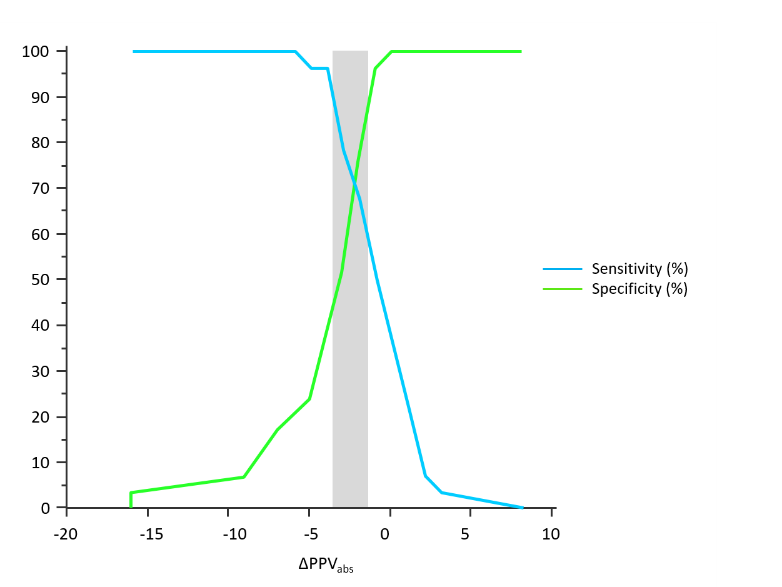


**Upper panel:** Receiver-operating characteristic curves expressing the ability of changes in pulse pressure variation, expressed in absolute value, during the PEEP-test to detect volume responsiveness.

**Bottom panel:** Sensitivity and specificity of the changes in pulse pressure variation induced by the PEEP-test depending on the test result. Gray zone represents an uncertain zone with cut-off values of -3.6 (sensitivity <90%) and -1.3 (specificity <90%).

∆PPV_abs_: change in pulse pressure variation in absolute value; PEEP: positive end-expiratory pressure

# Figure S8. Performance of changes in pulse pressure during the PEEP-test to detect volume responsiveness in patients with no atrial fibrillation (n=57)


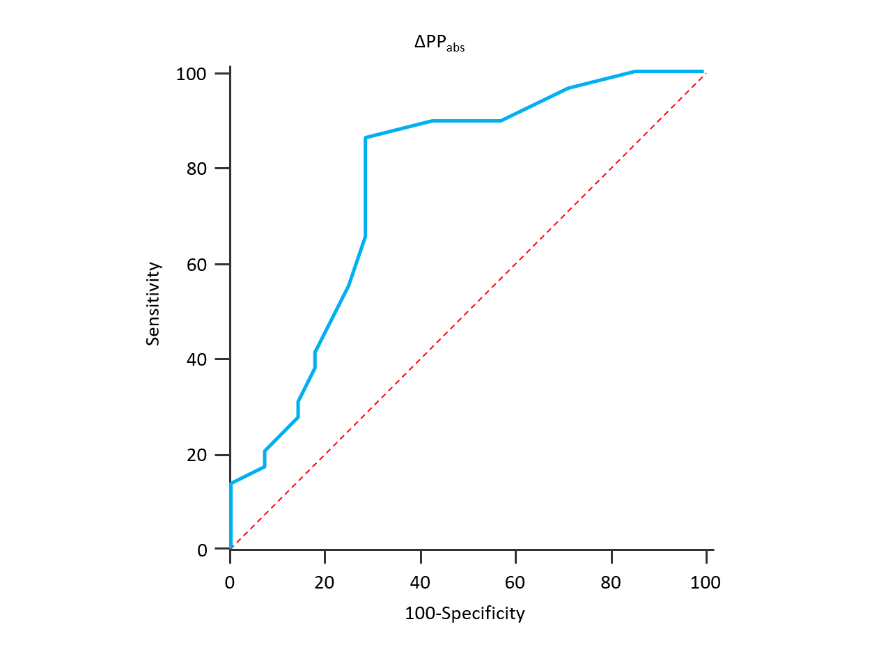


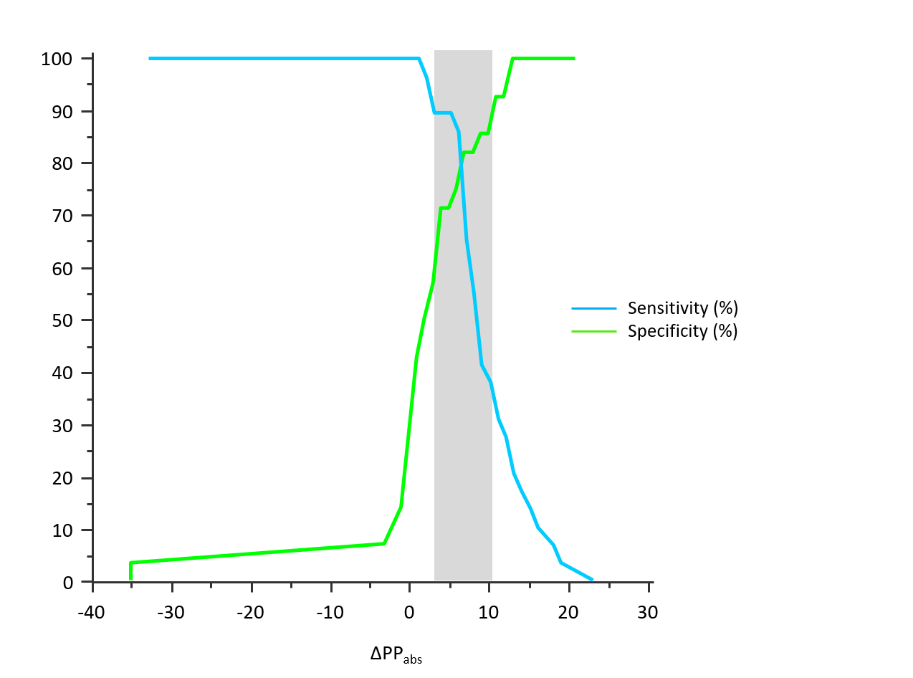


**Upper panel:** Receiver-operating characteristic curves expressing the ability of changes in pulse pressure, expressed in absolute value, during the PEEP-test to detect volume responsiveness.

**Bottom panel:** Sensitivity and specificity of the changes in pulse pressure induced by the PEEP-test depending on the test result. Gray zone represents an uncertain zone with cut-off values of 1.0 (sensitivity <90%) and 10.6 (specificity <90%).

∆PP_abs_: change in pulse pressure in absolute value; PEEP: positive end-expiratory pressure

# Figure S9. Receiver-operating characteristic curves expressing the performance of changes in cardiac index during the PEEP-test to detect volume responsiveness in low-recruiters (n=21) and high-recruiters (n=21) patients


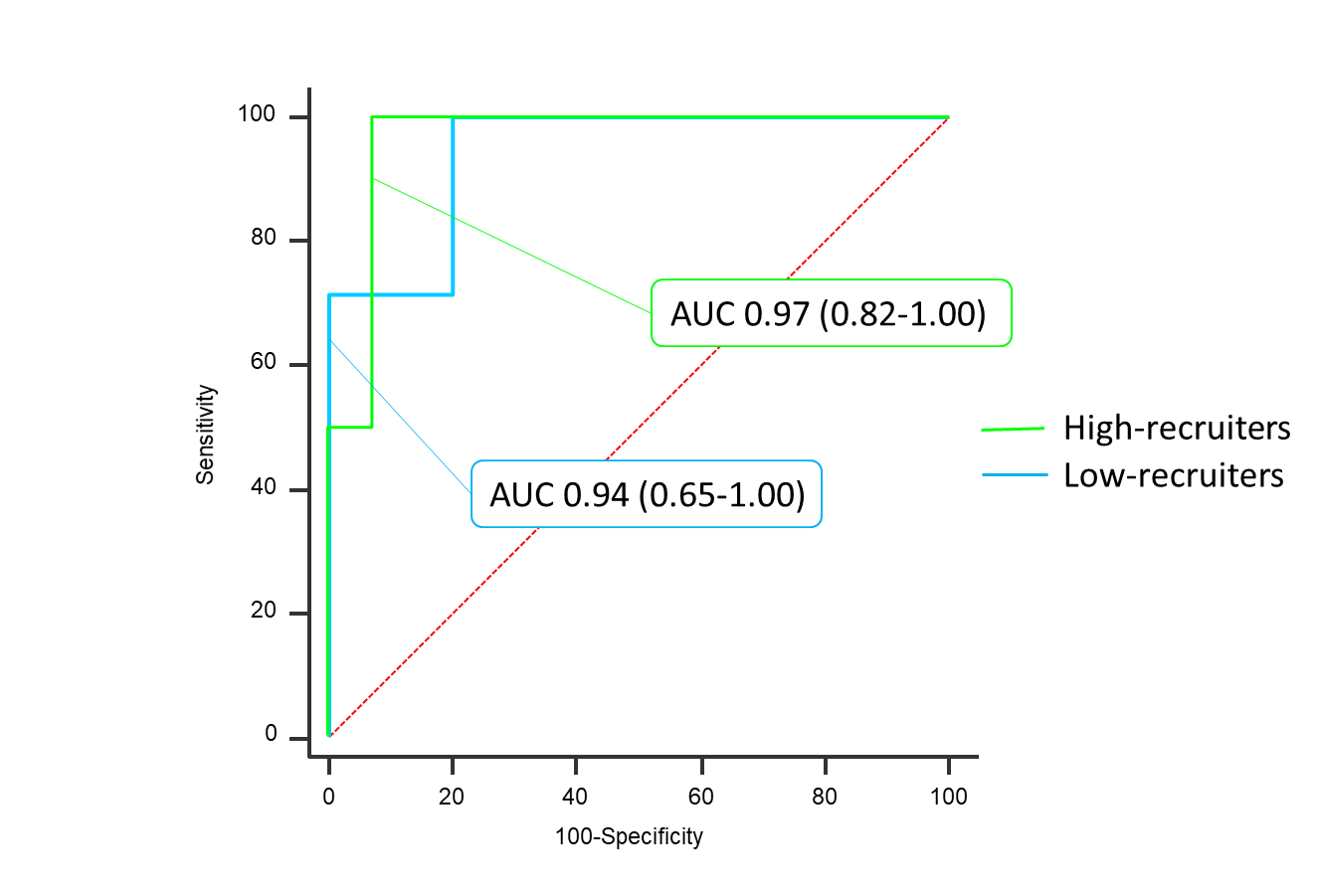


# Table S1. Hemodynamic variables during the study protocol in high recruiters and low recruiters (n=42)

|  | Baseline 1  (n=42) | PLR  (n=42) | Baseline 2 (n=42) | PEEP-test (n=42) | Baseline 3 (n=42) |
| --- | --- | --- | --- | --- | --- |
| HR, beats/min  *Low recruiters (n=21)*  *High recruiters (n=21)* | 71 ± 20  69 ± 13 | 70 ± 20  67 ± 12* | 71 ± 20  69 ± 12 | 70 ± 19  67 ± 12 | 71 ± 19  69 ± 12 |
| SAP, mmHg  *Low recruiters (n=21)*  *High recruiters (n=21)* | 119 ± 21  107 ± 23 | 131 ± 26*  122 ± 23* | 117 ± 21  107 ± 22 | 128 ± 26^¤^  117 ± 21^¤^ | 118 ± 20  106 ± 21 |
| DAP, mmHg  *Low recruiters (n=21)*  *High recruiters (n=21)* | 60 ± 10  53 ± 9 | 65 ± 12  59 ± 11* | 59 ± 11  52 ± 8 | 62 ± 11  54 ± 9^¤^ | 59 ± 10  51 ± 8 |
| MAP, mmHg  *Low recruiters (n=21)*  *High recruiters (n=21)* | 79 ± 12  70 ± 14 | 88 ± 16*  81 ± 15* | 78 ± 13  71 ± 13 | 85 ± 15^¤^  76 ± 13^¤^ | 79 ± 12  70 ± 12 |
| CVP, mmHg  *Low recruiters (n=21)*  *High recruiters (n=21)* | 8 ± 3  10 ± 3 | 11 ± 2*  14 ± 3* | 7 ± 2  9 ± 3 | 6 ± 2^¤^  8 ± 3^¤^ | 7 ± 3  10 ± 3 |
| CI, L/min/m^2^  *Low recruiters (n=21)*  *High recruiters (n=21)* | 2.4 ± 0.8  2.5 ± 0.8 | 2.7 ± 1.0*  2.6 ± 0.7* | 2.4 ± 0.8  2.5 ± 0.8 | 2.8 ± 0.9^¤^  2.8 ± 0.8^¤^ | 2.4 ± 0.8  2.5 ± 0.7 |
| PPV, %  *Low recruiters (n=21)*  *High recruiters (n=21)* | 7 ± 5  5 ± 3 | 4 ± 3*  5 ± 4 | 7 ± 4  5 ± 3 | 5 ± 4^¤^  4 ± 3^¤^ | 7 ± 5  5 ± 2 |
| SVV, %  *Low recruiters (n=21)*  *High recruiters (n=21)* | 7 ± 5  6 ± 4 | 5 ± 3*  5 ± 2 | 8 ± 5  6 ± 3 | 5 ± 4^¤^  6 ± 4^¤^ | 7 ± 4  6 ± 4 |

*p<0.05 PLR *vs.* Baseline 1

^¤^ p<0.05 PEEP-test *vs.* Baseline 2

^ⱡ^ p<0.05 Volume expansion *vs.* Baseline 3

^$^ p<0.05 High recruiters vs Low recruiters

CVP: central venous pressure; CI: cardiac index; DAP: diastolic arterial pressure; HR: heart rate; MAP: mean arterial pressure; PLR: passive leg raising; PPV: pulse pressure variation; SAP: systolic arterial pressure; SVV: stroke volume variation.

Values are expressed as mean ± standard deviation.

# Table S2. Variability of diagnostic accuracy of changes in cardiac index during PEEP-test to detect volume responsiveness

| Criterion | Sensitivity | 95% CI | Specificity | 95% CI | +LR | 95% CI | -LR | 95% CI | +PV | 95% CI | -PV | 95% CI |
| --- | --- | --- | --- | --- | --- | --- | --- | --- | --- | --- | --- | --- |
| >-0.0403 | 100.00 | 88.8 - 100.0 | 3.03 | 0.08 - 15.8 | 1.03 | 1.0 - 1.1 | 0.00 |  | 49.2 | 36.4 - 62.1 | 100.0 | 2.5 - 100.0 |
| >-0.03077 | 100.00 | 88.8 - 100.0 | 6.06 | 0.7 - 20.2 | 1.06 | 1.0 - 1.2 | 0.00 |  | 50.0 | 37.0 - 63.0 | 100.0 | 15.8 - 100.0 |
| >-0.0133 | 100.00 | 88.8 - 100.0 | 9.09 | 1.9 - 24.3 | 1.10 | 1.0 - 1.2 | 0.00 |  | 50.8 | 37.7 - 63.9 | 100.0 | 29.2 - 100.0 |
| >-0.0074 | 100.00 | 88.8 - 100.0 | 12.12 | 3.4 - 28.2 | 1.14 | 1.0 - 1.3 | 0.00 |  | 51.7 | 38.4 - 64.8 | 100.0 | 39.8 - 100.0 |
| >-0.0053 | 100.00 | 88.8 - 100.0 | 15.15 | 5.1 - 31.9 | 1.18 | 1.0 - 1.4 | 0.00 |  | 52.5 | 39.1 - 65.7 | 100.0 | 47.8 - 100.0 |
| >-0.0032 | 100.00 | 88.8 - 100.0 | 18.18 | 7.0 - 35.5 | 1.22 | 1.0 - 1.4 | 0.00 |  | 53.4 | 39.9 - 66.7 | 100.0 | 54.1 - 100.0 |
| >0.01 | 100.00 | 88.8 - 100.0 | 21.21 | 9.0 - 38.9 | 1.27 | 1.1 - 1.5 | 0.00 |  | 54.4 | 40.7 - 67.6 | 100.0 | 59.0 - 100.0 |
| >0.014 | 100.00 | 88.8 - 100.0 | 24.24 | 11.1 - 42.3 | 1.32 | 1.1 - 1.6 | 0.00 |  | 55.4 | 41.5 - 68.7 | 100.0 | 63.1 - 100.0 |
| >0.0271 | 100.00 | 88.8 - 100.0 | 27.27 | 13.3 - 45.5 | 1.37 | 1.1 - 1.7 | 0.00 |  | 56.4 | 42.3 - 69.7 | 100.0 | 66.4 - 100.0 |
| >0.0286 | 100.00 | 88.8 - 100.0 | 30.30 | 15.6 - 48.7 | 1.43 | 1.1 - 1.8 | 0.00 |  | 57.4 | 43.2 - 70.8 | 100.0 | 69.2 - 100.0 |
| >0.0366 | 100.00 | 88.8 - 100.0 | 33.33 | 18.0 - 51.8 | 1.50 | 1.2 - 1.9 | 0.00 |  | 58.5 | 44.1 - 71.9 | 100.0 | 71.5 - 100.0 |
| >0.0423 | 100.00 | 88.8 - 100.0 | 36.36 | 20.4 - 54.9 | 1.57 | 1.2 - 2.0 | 0.00 |  | 59.6 | 45.1 - 73.0 | 100.0 | 73.5 - 100.0 |
| >0.0448 | 100.00 | 88.8 - 100.0 | 39.39 | 22.9 - 57.9 | 1.65 | 1.3 - 2.2 | 0.00 |  | 60.8 | 46.1 - 74.2 | 100.0 | 75.3 - 100.0 |
| >0.0469 | 100.00 | 88.8 - 100.0 | 42.42 | 25.5 - 60.8 | 1.74 | 1.3 - 2.3 | 0.00 |  | 62.0 | 47.2 - 75.3 | 100.0 | 76.8 - 100.0 |
| >0.049 | 100.00 | 88.8 - 100.0 | 45.45 | 28.1 - 63.6 | 1.83 | 1.3 - 2.5 | 0.00 |  | 63.3 | 48.3 - 76.6 | 100.0 | 78.2 - 100.0 |
| >0.0552 | 100.00 | 88.8 - 100.0 | 48.48 | 30.8 - 66.5 | 1.94 | 1.4 - 2.7 | 0.00 |  | 64.6 | 49.5 - 77.8 | 100.0 | 79.4 - 100.0 |
| >0.0556 | 100.00 | 88.8 - 100.0 | 51.52 | 33.5 - 69.2 | 2.06 | 1.5 - 2.9 | 0.00 |  | 66.0 | 50.7 - 79.1 | 100.0 | 80.5 - 100.0 |
| >0.0596 | 100.00 | 88.8 - 100.0 | 54.55 | 36.4 - 71.9 | 2.20 | 1.5 - 3.2 | 0.00 |  | 67.4 | 52.0 - 80.5 | 100.0 | 81.5 - 100.0 |
| >0.0613 | 100.00 | 88.8 - 100.0 | 57.58 | 39.2 - 74.5 | 2.36 | 1.6 - 3.5 | 0.00 |  | 68.9 | 53.4 - 81.8 | 100.0 | 82.4 - 100.0 |
| >0.0662 | 100.00 | 88.8 - 100.0 | 60.61 | 42.1 - 77.1 | 2.54 | 1.7 - 3.9 | 0.00 |  | 70.5 | 54.8 - 83.2 | 100.0 | 83.2 - 100.0 |
| >0.0685 | 100.00 | 88.8 - 100.0 | 63.64 | 45.1 - 79.6 | 2.75 | 1.8 - 4.3 | 0.00 |  | 72.1 | 56.3 - 84.7 | 100.0 | 83.9 - 100.0 |
| >0.0708 | 100.00 | 88.8 - 100.0 | 66.67 | 48.2 - 82.0 | 3.00 | 1.9 - 4.9 | 0.00 |  | 73.8 | 58.0 - 86.1 | 100.0 | 84.6 - 100.0 |
| >0.0732 | 100.00 | 88.8 - 100.0 | 69.70 | 51.3 - 84.4 | 3.30 | 2.0 - 5.5 | 0.00 |  | 75.6 | 59.7 - 87.6 | 100.0 | 85.2 - 100.0 |
| >0.0772 | 100.00 | 88.8 - 100.0 | 72.73 | 54.5 - 86.7 | 3.67 | 2.1 - 6.4 | 0.00 |  | 77.5 | 61.5 - 89.2 | 100.0 | 85.8 - 100.0 |
| >0.08 | 96.77 | 83.3 - 99.9 | 72.73 | 54.5 - 86.7 | 3.55 | 2.0 - 6.2 | 0.044 | 0.006 - 0.3 | 76.9 | 60.7 - 88.9 | 96.0 | 79.6 - 99.9 |
| >0.0802 | 96.77 | 83.3 - 99.9 | 75.76 | 57.7 - 88.9 | 3.99 | 2.2 - 7.3 | 0.043 | 0.006 - 0.3 | 78.9 | 62.7 - 90.4 | 96.2 | 80.4 - 99.9 |
| >0.082 | 96.77 | 83.3 - 99.9 | 78.79 | 61.1 - 91.0 | 4.56 | 2.4 - 8.8 | 0.041 | 0.006 - 0.3 | 81.1 | 64.8 - 92.0 | 96.3 | 81.0 - 99.9 |
| >0.083 | 96.77 | 83.3 - 99.9 | 81.82 | 64.5 - 93.0 | 5.32 | 2.6 - 11.0 | 0.039 | 0.006 - 0.3 | 83.3 | 67.2 - 93.6 | 96.4 | 81.7 - 99.9 |
| >0.086 | 96.77 | 83.3 - 99.9 | 84.85 | 68.1 - 94.9 | 6.39 | 2.8 - 14.4 | 0.038 | 0.005 - 0.3 | 85.7 | 69.7 - 95.2 | 96.6 | 82.2 - 99.9 |
| >0.0864 | 93.55 | 78.6 - 99.2 | 84.85 | 68.1 - 94.9 | 6.17 | 2.7 - 13.9 | 0.076 | 0.02 - 0.3 | 85.3 | 68.9 - 95.0 | 93.3 | 77.9 - 99.2 |
| >0.0874 | 90.32 | 74.2 - 98.0 | 84.85 | 68.1 - 94.9 | 5.96 | 2.6 - 13.5 | 0.11 | 0.04 - 0.3 | 84.8 | 68.1 - 94.9 | 90.3 | 74.2 - 98.0 |
| >0.0884 | 87.10 | 70.2 - 96.4 | 84.85 | 68.1 - 94.9 | 5.75 | 2.5 - 13.0 | 0.15 | 0.06 - 0.4 | 84.4 | 67.2 - 94.7 | 87.5 | 71.0 - 96.5 |
| >0.0902 | 83.87 | 66.3 - 94.5 | 84.85 | 68.1 - 94.9 | 5.54 | 2.4 - 12.6 | 0.19 | 0.08 - 0.4 | 83.9 | 66.3 - 94.5 | 84.8 | 68.1 - 94.9 |
| >0.0918 | 80.65 | 62.5 - 92.5 | 84.85 | 68.1 - 94.9 | 5.32 | 2.3 - 12.2 | 0.23 | 0.1 - 0.5 | 83.3 | 65.3 - 94.4 | 82.4 | 65.5 - 93.2 |
| >0.1025 | 80.65 | 62.5 - 92.5 | 87.88 | 71.8 - 96.6 | 6.65 | 2.6 - 16.9 | 0.22 | 0.1 - 0.5 | 86.2 | 68.3 - 96.1 | 82.9 | 66.4 - 93.4 |
| >0.103 | 77.42 | 58.9 - 90.4 | 87.88 | 71.8 - 96.6 | 6.39 | 2.5 - 16.3 | 0.26 | 0.1 - 0.5 | 85.7 | 67.3 - 96.0 | 80.6 | 64.0 - 91.8 |
| >0.1178 | 74.19 | 55.4 - 88.1 | 87.88 | 71.8 - 96.6 | 6.12 | 2.4 - 15.7 | 0.29 | 0.2 - 0.5 | 85.2 | 66.3 - 95.8 | 78.4 | 61.8 - 90.2 |
| >0.1182 | 74.19 | 55.4 - 88.1 | 90.91 | 75.7 - 98.1 | 8.16 | 2.7 - 24.5 | 0.28 | 0.2 - 0.5 | 88.5 | 69.8 - 97.6 | 78.9 | 62.7 - 90.4 |
| >0.1194 | 70.97 | 52.0 - 85.8 | 90.91 | 75.7 - 98.1 | 7.81 | 2.6 - 23.5 | 0.32 | 0.2 - 0.6 | 88.0 | 68.8 - 97.5 | 76.9 | 60.7 - 88.9 |
| >0.1326 | 67.74 | 48.6 - 83.3 | 90.91 | 75.7 - 98.1 | 7.45 | 2.5 - 22.5 | 0.35 | 0.2 - 0.6 | 87.5 | 67.6 - 97.3 | 75.0 | 58.8 - 87.3 |
| >0.1328 | 64.52 | 45.4 - 80.8 | 90.91 | 75.7 - 98.1 | 7.10 | 2.3 - 21.5 | 0.39 | 0.2 - 0.6 | 87.0 | 66.4 - 97.2 | 73.2 | 57.1 - 85.8 |
| >0.1402 | 61.29 | 42.2 - 78.2 | 90.91 | 75.7 - 98.1 | 6.74 | 2.2 - 20.6 | 0.43 | 0.3 - 0.7 | 86.4 | 65.1 - 97.1 | 71.4 | 55.4 - 84.3 |
| >0.1437 | 58.06 | 39.1 - 75.5 | 90.91 | 75.7 - 98.1 | 6.39 | 2.1 - 19.6 | 0.46 | 0.3 - 0.7 | 85.7 | 63.7 - 97.0 | 69.8 | 53.9 - 82.8 |
| >0.1465 | 54.84 | 36.0 - 72.7 | 90.91 | 75.7 - 98.1 | 6.03 | 2.0 - 18.6 | 0.50 | 0.3 - 0.7 | 85.0 | 62.1 - 96.8 | 68.2 | 52.4 - 81.4 |
| >0.1474 | 54.84 | 36.0 - 72.7 | 93.94 | 79.8 - 99.3 | 9.05 | 2.3 - 36.0 | 0.48 | 0.3 - 0.7 | 89.5 | 66.9 - 98.7 | 68.9 | 53.4 - 81.8 |
| >0.1477 | 51.61 | 33.1 - 69.8 | 93.94 | 79.8 - 99.3 | 8.52 | 2.1 - 34.0 | 0.52 | 0.4 - 0.7 | 88.9 | 65.3 - 98.6 | 67.4 | 52.0 - 80.5 |
| >0.1565 | 48.39 | 30.2 - 66.9 | 93.94 | 79.8 - 99.3 | 7.98 | 2.0 - 32.1 | 0.55 | 0.4 - 0.8 | 88.2 | 63.6 - 98.5 | 66.0 | 50.7 - 79.1 |
| >0.1571 | 48.39 | 30.2 - 66.9 | 96.97 | 84.2 - 99.9 | 15.97 | 2.2 - 113.8 | 0.53 | 0.4 - 0.8 | 93.7 | 69.8 - 99.8 | 66.7 | 51.6 - 79.6 |
| >0.1611 | 45.16 | 27.3 - 64.0 | 96.97 | 84.2 - 99.9 | 14.90 | 2.1 - 106.7 | 0.57 | 0.4 - 0.8 | 93.3 | 68.1 - 99.8 | 65.3 | 50.4 - 78.3 |
| >0.1726 | 45.16 | 27.3 - 64.0 | 100.00 | 89.4 - 100.0 |  |  | 0.55 | 0.4 - 0.8 | 100.0 | 76.8 - 100.0 | 66.0 | 51.2 - 78.8 |
| >0.1727 | 41.94 | 24.5 - 60.9 | 100.00 | 89.4 - 100.0 |  |  | 0.58 | 0.4 - 0.8 | 100.0 | 75.3 - 100.0 | 64.7 | 50.1 - 77.6 |
| >0.1758 | 38.71 | 21.8 - 57.8 | 100.00 | 89.4 - 100.0 |  |  | 0.61 | 0.5 - 0.8 | 100.0 | 73.5 - 100.0 | 63.5 | 49.0 - 76.4 |
| >0.179 | 35.48 | 19.2 - 54.6 | 100.00 | 89.4 - 100.0 |  |  | 0.65 | 0.5 - 0.8 | 100.0 | 71.5 - 100.0 | 62.3 | 47.9 - 75.2 |
| >0.1818 | 32.26 | 16.7 - 51.4 | 100.00 | 89.4 - 100.0 |  |  | 0.68 | 0.5 - 0.9 | 100.0 | 69.2 - 100.0 | 61.1 | 46.9 - 74.1 |
| >0.184 | 29.03 | 14.2 - 48.0 | 100.00 | 89.4 - 100.0 |  |  | 0.71 | 0.6 - 0.9 | 100.0 | 66.4 - 100.0 | 60.0 | 45.9 - 73.0 |
| >0.2006 | 25.81 | 11.9 - 44.6 | 100.00 | 89.4 - 100.0 |  |  | 0.74 | 0.6 - 0.9 | 100.0 | 63.1 - 100.0 | 58.9 | 45.0 - 71.9 |
| >0.2009 | 22.58 | 9.6 - 41.1 | 100.00 | 89.4 - 100.0 |  |  | 0.77 | 0.6 - 0.9 | 100.0 | 59.0 - 100.0 | 57.9 | 44.1 - 70.9 |
| >0.2161 | 19.35 | 7.5 - 37.5 | 100.00 | 89.4 - 100.0 |  |  | 0.81 | 0.7 - 1.0 | 100.0 | 54.1 - 100.0 | 56.9 | 43.2 - 69.8 |
| >0.2232 | 16.13 | 5.5 - 33.7 | 100.00 | 89.4 - 100.0 |  |  | 0.84 | 0.7 - 1.0 | 100.0 | 47.8 - 100.0 | 55.9 | 42.4 - 68.8 |
| >0.2238 | 12.90 | 3.6 - 29.8 | 100.00 | 89.4 - 100.0 |  |  | 0.87 | 0.8 - 1.0 | 100.0 | 39.8 - 100.0 | 55.0 | 41.6 - 67.9 |
| >0.2897 | 9.68 | 2.0 - 25.8 | 100.00 | 89.4 - 100.0 |  |  | 0.90 | 0.8 - 1.0 | 100.0 | 29.2 - 100.0 | 54.1 | 40.8 - 66.9 |
| >0.3286 | 6.45 | 0.8 - 21.4 | 100.00 | 89.4 - 100.0 |  |  | 0.94 | 0.9 - 1.0 | 100.0 | 15.8 - 100.0 | 53.2 | 40.1 - 66.0 |
| >0.4145 | 3.23 | 0.08 - 16.7 | 100.00 | 89.4 - 100.0 |  |  | 0.97 | 0.9 - 1.0 | 100.0 | 2.5 - 100.0 | 52.4 | 39.4 - 65.1 |

CI: confidence interval; LR: likelihood ratio; PV: predictive value
